# Chemical Characterization and Antimicrobial Activity of Green Propolis from the Brazilian Caatinga Biome

**DOI:** 10.3390/plants13243576

**Published:** 2024-12-21

**Authors:** Jennyfer A. Aldana-Mejía, Victor Pena Ribeiro, Kumar Katragunta, Bharathi Avula, Kiran Kumar Tatapudi, Jairo Kenupp Bastos, Ikhlas A. Khan, Kumudini Meepagala, Samir A. Ross

**Affiliations:** 1 National Center for Natural Products Research, School of Pharmacy, University of Mississippi, University, MS 38677, USA; jaaldana@olemiss.edu (J.A.A.-M.); kkatragu@olemiss.edu (K.K.); bavula@olemiss.edu (B.A.); kktatapu@olemiss.edu (K.K.T.); ikhan@olemiss.edu (I.A.K.); 2 Agricultural Research Service, Natural Products Utilization Research Unit, U.S. Department of Agriculture, University, MS 38677, USA; victor.ribeiro@usda.gov (V.P.R.); kumudini.meepagala@usda.gov (K.M.); 3 School of Pharmaceutical Sciences of Ribeirão Preto, University of São Paulo, Av. do Café, Ribeirão Preto 14040-930, Brazil; jkbastos@fcfrp.usp.br; 4 Division of Pharmacognosy, Department of BioMolecular Sciences, School of Pharmacy, University of Mississippi, University, MS 38677, USA

**Keywords:** Brazilian green propolis, flavonoids, chemical profiling, antimicrobial activity, methicillin-resistant *Staphylococcus aureus* (MRSA), vancomycin-resistant *Enterococcus faecium* (VRE), Caatinga biome

## Abstract

Green propolis, particularly from the unique flora of the Brazilian Caatinga biome, has gained significant interest due to its diverse chemical composition and biological activities. This study focuses on the chemical characterization and antimicrobial evaluation of Caatinga green propolis. Twelve compounds were isolated through different chromatographic techniques, including flavanones (naringenin, 7-*O*-methyleriodictyol, sakuranetin), flavones (hispidulin, cirsimaritin), flavonols (quercetin, quercetin-3-methyl ether, kaempferol, 6-methoxykaempferol, viscosine, penduletin), and one chalcone (kukulkanin B). Using liquid chromatography–quadrupole time-of-flight tandem mass spectrometry (LC-QToF-MS), a total of 55 compounds excluding reference standards were tentatively identified, which include flavonoids, phenolic acids derivatives, and alkaloids, with flavonols, flavanones, and flavones being predominant. Antimicrobial testing against pathogens revealed that the crude extract exhibited low inhibitory activity, against Gram-positive bacteria, including methicillin-resistant *Staphylococcus aureus* (MRSA) and vancomycin-resistant *Enterococcus faecium* (VRE) (IC_50_: 148.4 and 120.98 µg/mL, respectively). Although the isolated compounds showed limited individual activity, a fraction containing sakuranetin and penduletin (Fraction 8) exhibited moderated activity against *Cryptococcus neoformans* (IC_50_: 47.86 µg/mL), while a fraction containing quercetin and hispidulin showed moderated activity against VRE (IC_50_: 16.99 µg/mL). These findings highlight the potential application of Caatinga green propolis as an antimicrobial agent, particularly against resistant bacterial strains, and underscore the importance of synergistic interactions between compounds in enhancing biological effects.

## 1. Introduction

The use of natural products as pharmaceuticals dates back to ancient times and continues to be a significant area of research for developing alternatives that modulate several diseases with minimal complications [1]. Among these natural products, propolis—a resinous material collected by bees from plant buds and exudates, mixed with wax from their glands—has garnered significant attention [2,3,4].

Propolis has a critical biological function within bee colonies, including lining the inner walls of hives, sealing cracks and openings in the nest, and protecting the hive entrance from environmental factors and intruders [2,3]. These roles help reduce the risk of fungal and bacterial growth, as well as the transmission of diseases and parasites throughout the colony [3,4]. Beyond its role in the hive, propolis is valued for its medicinal properties, including its use as an antiseptic to treat wounds, skin lesions, ulcers, and to alleviate swelling and pain [4,5].

The chemical composition, aroma, and color of propolis are highly variable and depend on its botanical source and the season [6]. In Brazil, different types of propolis have been identified based on organoleptic characteristics, chemical composition, and geographic location [7]. The Brazilian propolis market has primarily focused on green propolis from the southeast, brown propolis from the central–south, and red propolis from the northeast. However, there has been growing interest in other types produced on a smaller scale. One such type is green propolis from the northeast of Brazil, specifically from the Caatinga biome [8]. This propolis is derived from *Mimosa tenuiflora* (Leguminosae, Mimosoideae), known as “jurema preta”, a plant whose bark has historically been utilized by African and Amerindian cultures [8,9].

Research suggests that the green coloration of Brazilian propolis, including that from the Caatinga and southeastern regions, results from chlorophylls derived from the aerial parts of plants [8]. Southeastern green propolis is characterized by prenylated phenylpropanoids (artepillin C, baccharin, and drupanin), hydroxycinnamic acids (*p*-coumaric acid), and chlorogenic acids (3,3-dicaffeoylquinic acid), along with flavonoids (kaempferide) [8]. In contrast, green propolis from the Caatinga region is distinguished by its predominant flavonoid composition, which contributes to its strong antioxidant properties [9]. Despite this, studies characterizing Caatinga green propolis remain limited. To date, some compounds, including flavonoids and chalcones such as quercetin, naringenin, isorhamnetin, violanthin, myricetin, dimethoxyquercetin, and dihydroxy-dimethoxychalcone, have been identified in this regional propolis [9,10]. Nevertheless, most research has focused on confirming its botanical origin rather than providing a comprehensive understanding of this complex natural matrix.

Numerous studies have explored the biological properties of green propolis from the Caatinga and its components, revealing its antioxidant [9,10,11,12], anti-HIV [11], leishmanicidal [13], immunomodulatory [14], anti-inflammatory and antibacterial [12], and cytotoxic activities [15]. Propolis and its constituents exhibit significant antimicrobial effects [8] and may synergize with antimicrobial drugs, highlighting their therapeutic potential and value for pharmaceutical innovation [16]. The global rise in antimicrobial-resistant strains, such as methicillin-resistant *S. aureus* (MRSA) and vancomycin-resistant *Enterococcus* (VRE), has intensified the need for innovative treatment strategies. Notably, VRE serves as a reservoir of vancomycin resistance for other pathogens, including MRSA [17]. Ethanolic plant extracts rich in phenolic compounds have demonstrated effective in vitro activity against MRSA, highlighting propolis as a promising alternative for antimicrobial development [18].

The primary objective of this study is to characterize the chemical constituents of Brazilian green propolis through the isolation of its compounds and mass spectrometry analysis, and to assess its antimicrobial activity. Understanding the chemical composition and biological activity of Brazilian green propolis is crucial for evaluating its potential and standardizing its future use in pharmacological applications. To integrate northeast Brazilian propolis into the apiculture market, several steps are essential, including the establishment of standard parameters, chemical profile analysis, and biological activity evaluation [9]. A comprehensive understanding of its complex chemical composition and potent biological properties will support the development of innovative natural therapies and provide scientific validation for its traditional uses.

## 2. Results

### 2.1. Chemical Characterization

**Figure 1:**
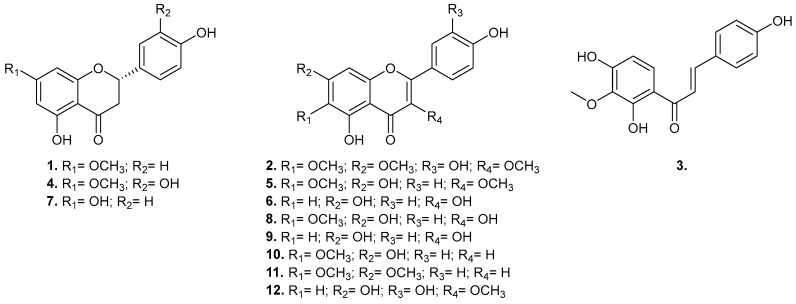
Chemical structures of the isolated constituents of the Brazilian green propolis of Caatinga extract: sakuranetin (**1**), penduletin (**2**), kukulkanin B (**3**), 7-*O*-methyleriodictyol (**4**), viscosine (**5**), kaempferol (**6**), naringenin (**7**), 6-methoxykaempferol (**8**), quercetin (**9**), hispidulin (**10**), cirsimaritin (**11**), and quercetin-3-methyl ether (**12**).

Green propolis has garnered significant attention due to its diverse biological activities, primarily attributed to its complex chemical composition. In this study, we investigated the chemical profile of green propolis from the Caatinga, identifying several key compounds. Our analysis revealed a rich presence of flavonoids including flavonols (quercetin, quercetin-3-methyl ether, kaempferol, 6-methoxykaempferol, viscosine, penduletin), flavones (hispidulin, cirsimaritin), flavanones (naringenin, 7-*O*-methyleriodictyol, sakuranetin), and one chalcone (kukulkanin B, Figure 1).

### 2.2. Phytochemical Screening by LC-QToF-MS

**Figure 2:**
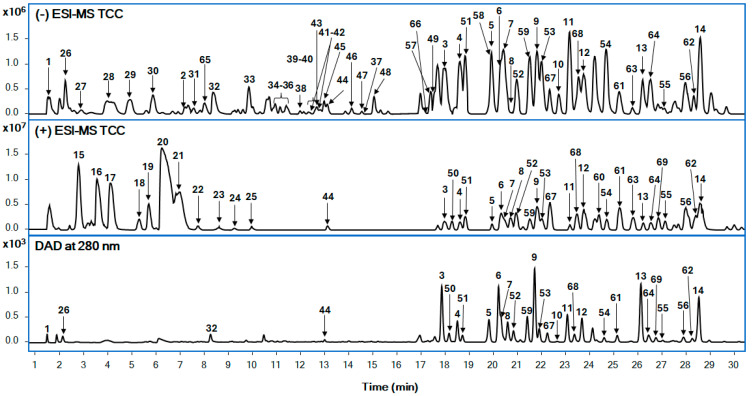
Total compound chromatogram (TCC—positive and negative modes) and LC-DAD at 280 nm for Brazilian green propolis of Caatinga extract.

The chemical constituents of the Brazilian green propolis of Caatinga were analyzed using reverse phase liquid chromatography in gradient elution mode followed by the identification of secondary metabolites using QToF-MS analysis in hyphenation. Here, we have used electrospray ionization–high-resolution mass spectrometry (ESI-QToF-MS). The LC-QToF-MS method facilitates the tentative identification of the compounds, based on their exact mass, fragment ions, and retention patterns. Respective LC-DAD chromatograms were presented at 280 nm (Figure 2) along with total current chromatograms (TCC) in both positive and negative ionization modes, to provide a comparative chromatographic profile. Data analyses were conducted in both modes to ensure the identification of the compounds and to confirm the better ionization of the secondary metabolites from the propolis extract.

The identification of compounds using the LC-QToF-MS method of analysis in this study was divided into two levels: identification through comparison with reference standards (Compounds **1**–**14**) and tentative identification using the mass spectral data and the previously reported literature cited in Table 1. The phytochemical screening included the identification of phenolic acids and hydroxybenzoic acid derivatives (**1**–**2**, **26**–**33**), flavonols (**3**, **4**, **7**–**9**, **14**, **34**–**56**), flavanones (**5**, **11**, **13**, **66**–**67**), flavones (**6**, **12**, **57**–**64**), alkaloids (**15**–**25**), and other phenolic compounds (**10**, **65**, **68**, **69**), as presented in the table.

#### 2.2.1. Phenolic Acids and Hydroxybenzoic Acid Derivatives (**1**–**2**, **26**–**33**)

A total of ten compounds of this class were identified based on their characteristic fragmentation patterns. Quinic acid (**1**) displays fragmentation behavior typical of phenolic acids. In negative ion mode, quinic acid presents the parent ion [M−H]^−^ at *m*/*z* 191.0558, with common water loss fragments observed at *m*/*z* 173.0455 and 155.0349. These patterns reflect the high degree of hydroxylation characteristic of this class. Caffeic (**2**) acid exhibits similar patterns, with [M−H]^−^ ions at *m*/*z* 179.0349, and its fragmentation includes losses of H_2_O (*m*/*z* 161.0027) and CO_2_ (*m*/*z* 135.0452).

In the negative ion mode, gallic acid (**26**) shows a parent ion at *m*/*z* 169.0143, which undergoes decarboxylation, resulting in a fragment at *m*/*z* 125.0241. This pattern reflects its simpler structure and high aromaticity, a characteristic commonly found in hydroxybenzoic acids. Other gallic acid derivatives were tentatively identified as presented in Table 1.

#### 2.2.2. Flavonols (**3**, **4**, **7**–**9**, **14**, **34**–**56**)

These compounds were the most abundant constituents in the green propolis sample. Among the isolated compounds, quercetin (**3**) exhibited a fragmentation pattern in positive ion mode, characterized by typical losses of CH_3_, H_2_O, and CO groups, resulting in fragments at *m*/*z* 285.0544, 257.0445, and 229.0478. Similarly, kaempferol (**7**), with a precursor ion at 287.0567 at the positive mode, undergoes fragmentation with the loss of H_2_O and CO groups, yielding fragments at *m*/*z* 241.0412 and 231.0475. Other methoxylated flavonols, including quercetin-3-methyl ether (**4**), 6-methoxykaempferol (**8**), viscosine (**9**), and penduletin (**14**), demonstrated similar fragmentation patterns with CH_3_ group losses in both positive and negative ion modes. Several derivatives of kaempferol, quercetin, and pinobanksin were tentatively identified in the Caatinga green propolis sample, underscoring the predominance of methoxylated and hydroxylated flavonoid compounds characteristic of this type of propolis.

#### 2.2.3. Flavanones (**5**, **11**, **13**, **66**–**67**)

Five flavanones were identified based on the characteristic fragments observed, respective to the precursor ions observed. Naringenin (**5**) one of the isolated compounds shows *m*/*z* 273.0756 precursor ion in the positive mode with substantial fragment ions at *m*/*z* 269.0472 and 241.0526, via dehydration and decarbonylation. Also, 7-*O*-methyleriodictyol (**11**), has a [M−H]^⁻^ precursor ion at *m*/*z* 301.0720, with resulting ions at *m*/*z* 165.0193 and 135.0450 due to the loss of a C_8_H_6_O_4_ and C_8_H_8_O_2_ groups, respectively, probably involving cleavage of the flavonoids’ B-ring and part of the C-ring, respectively. Sakuranetin (**13**) exhibits a [M−H]^−^ precursor ion at *m*/*z* 285.0770, with fragments arising from the cleavage of its methoxy group at *m*/*z* 270.0533. This leads to secondary ions typically observed at *m*/*z* 165.0186 and 119.0496, highlighting the methoxy functional group’s influence on the stability and breakdown of the molecule under negative ionization conditions.

#### 2.2.4. Flavones (**6**, **12**, **57**–**64**)

A total of ten flavones were identified in the green propolis extract, with most being methylated flavones, as confirmed by their fragmentation patterns. Among the isolated standards, hispidulin (**6**), with a [M−H]^−^ precursor ion at 299.0554, undergoes a loss of a CH_3_ group, resulting in a fragment at *m*/*z* 284.0325; further fragmentation with a *m*/*z* 117.0438 indicates the loss of a C_8_H_6_O_5_ moiety from parent moiety, corresponding to the retro-Diels–Alder (RDA) cleavage of the flavonoid skeleton. Cirsimaritin (**12**), which differs by a methoxy group at position 7 from the previous compound, displayed a [M−H]^−^ precursor ion at 313.0719, with fragment ions at *m*/*z* 298.0469 and 283.0246, indicating the loss of one or two CH_3_ groups. Other methoxylated flavones, such as luteolin methyl-ether (**59**), dihydroxy-trimethoxy flavone isomers (**60**–**62**), and acacetin (**64**), exhibited similar fragmentation patterns, characterized by the sequential loss of CH_3_ groups and carbonyl groups (CO), which is common in compounds with keto-enol systems or flavonoids with conjugated structures.

#### 2.2.5. Alkaloids (**15**–**25**)

Ten compounds were tentatively identified as alkaloids. Dimethyltryptamine *N*-oxide (**15**) exhibited a molecular ion at *m*/*z* 205.1325 at the positive mode, its fragmentation profile includes diagnostic ions at *m*/*z* 191.1170, indicating cleavage adjacent to the dimethyl group and *m*/*z* 173.1090, characteristic of *N*-oxide derivatives dehydration. Phenethylamine (**16**) showed a positive mode ion at *m*/*z* 122.0954, with prominent fragments at *m*/*z* 105.0712 due to the loss of a methylamine group, and *m*/*z* 77.0405, reflecting cleavage of the side chain. Methylphenethylamine (**17**), with a precursor ion at *m*/*z* 136.1127 at the positive mode, produced fragment ions at *m*/*z* 105.0726 and *m*/*z* 77.0403, following a fragmentation pathway similar to phenethylamine but with methyl substitution. The methyltryptamine (**20**) fragmentation pattern showed an [M+H]^+^ ion at *m*/*z* 175.1216, with fragmentation ions such as *m*/*z* 144.0815 and *m*/*z* 142.0665, indicating cleavage near the methyl-substituted amine group with subsequent loss of H_2_.

#### 2.2.6. Others (**10**, **65**, **68**, **69**)

Kukulkanin B (**10**), with a precursor ion peak [M+H]^−^ at *m*/*z* 285.0775, exhibits fragment ions at *m*/*z* 119.0506 and 165.0201. The ion at *m*/*z* 119 is characteristic of the B-ring cleavage, where a portion of the flavonoid is eliminated; the ion at *m*/*z* 165 indicates cleavage of a methylated portion of the B-ring or the C-ring, including a methoxy group.

A flava-3-ol was also detected as epigallocatechin gallate (**65**), with a precursor ion of 459.0913 at the positive mode, and fragmentation ions at *m*/*z* 289.0694, by the cleavage of the ester bond that releases the gallate moiety, leaving the core (epi)gallocatechin structure. One pterocarpan identified as melilotocarpan D (**68**), presented a precursor ion of 317.1017 at the positive mode with fragmentation ions at *m*/*z* 197.0442, representing the hydroxybenzoic acid fragment or a similar stable aromatic substructure derived from the A-ring, and 182.0198, arises from further fragmentation of the 197.0442 ion, specifically the loss of a methyl group, often from a methoxy substituent. One isoflavone was identified in the sample. Dihydroxy-dimethoxyisoflavone (**69**), in positive mode, shows a precursor ion of 315.0869, and fragments at *m*/*z* 301.0739 (loss of CH_2_) and 285.0780 likely due to the loss of a methoxy group as formaldehyde (CH_2_O).

### 2.3. Antimicrobial Assessment

The results presented in Table 2 demonstrate the antimicrobial efficacy of the Brazilian green propolis from Caatinga extract and its compounds against the microbial strains that demonstrate positive results. The propolis extract exhibits moderate activity, particularly against *C. neoformans* (192.50 µg/mL), MRS (148.44 µg/mL), and VRE (120.98 µg/mL). Such values suggest that, while propolis extract may not exert strong inhibitory effects on all pathogens tested, it shows moderate potential against certain Gram-positive bacteria, particularly in the case of resistant strains like MRS and VRE.

In the case of fraction F8, which consists of a refined extract from the original propolis, an increased inhibitory effect was noted against *C. neoformans* (47.86 µg/mL), MRS (142.34 µg/mL), and VRE (101.89 µg/mL). This enhanced efficacy, particularly against *C. neoformans*, could be attributed to the higher concentration of specific active compounds within this fraction, such as sakuranetin and penduletin. Fraction 14 displayed the most significant antimicrobial activity, especially with a MIC of 16.99 µg/mL against VRE, indicating it as a promising candidate for further study against drug-resistant bacteria. From this fraction, hispidulin and quercetin were isolated. In contrast, individual compounds exhibited minimum inhibitory concentrations (MICs) greater than 20 µg/mL for all tested strains.

## 3. Discussion

One of the initial studies on green propolis from the Brazilian Caatinga analyzed two samples from the state of Rio Grande do Norte and compared their chemical profiles with that of a *M. tenuiflora* sample [9]. Using LC-DAD-MS/MS, it was observed that the samples exhibited a high degree of similarity, indicating that *M. tenuiflora* is the botanical source of green propolis in the Caatinga biome. The major compounds identified in this new propolis type included quercetin-dimethyl ether, dihydroxy-trimethoxyflavone, kaempferol-methyl ether, quercetin-methyl ether, trihydroxy-dimethoxy chalcone, and dihydroxy-methoxy flavanone [9,10].

Gallic acid, syringic acid, *p*-coumaric acid, and caffeic acid have also been detected in ethanolic extracts of *M. tenuiflora* [26]. Caffeic acid and *p*-coumaric acid are also characteristic of Brazilian green propolis from Ceara state [11]. Additionally, *p*-coumaric acid is recognized as one of the chemical markers of Brazilian green propolis produced in the Southeast region [27].

Sakuranetin, penduletin, viscosine, quercetin-3-methyl ether, and cirsimaritin have been identified in *M. tenuiflora* and isolated from Brazilian green propolis from Bahia extract [28,29], while naringenin has been isolated from green propolis extract from Ceará State [11]. Recent studies conducted with green propolis samples from the Caatinga have succeeded in isolating and identifying more than eleven flavonols, four flavanones, and one prenylated flavanone [28]. The compounds santin, axillarin, viscosine, tamarixetin, and kaempferide were identified as the major constituents in the analyzed samples. These compounds were isolated for the first time in propolis samples, although some had previously been identified in samples from *Mimosa* genus species. However, compounds such as 6-methoxykaempferol and 7-*O*-methyleriodictyol are reported here for the first time in this type of propolis. This highlights the unique chemical profile of the sample analyzed and adds new data to the growing knowledge of the chemical diversity of green propolis from this region.

Several derivatives of kaempferide, penduletin, and pinobanksin were tentatively identified in the Caatinga green propolis sample, underscoring the predominance of methoxylated and hydroxylated flavonoid compounds characteristic of this type of propolis. Compounds such as quercetin-3-methyl ether, viscosine, and kaempferol, as well as penduletin, have previously been reported in Caatinga green propolis, along with other flavonols such as santin, ermanin, and axillarin [28]. Additionally, kaempferide and isokaempferide, which were tentatively identified in this study, have been noted as typical constituents of this propolis type [28]. Kukulkanin B, previously reported in the botanical source of this propolis, *M. tenuiflora*, collected in Mexico [30], is isolated in this study for the first time from Brazilian green propolis, highlighting the significant addition of chalcones to its chemical profile. Meanwhile, other chalcones such as the trihydroxy methoxy chalcone have been documented in the apices of the plant and in green propolis samples from the state of Rio Grande do Norte in Brazil [9].

The presence of alkaloids in the green propolis sample from the Caatinga region is also correlated with *M. tenuiflora* as its botanical source. Notably, *N*, *N*-dimethyltryptamine (DMT) and its *N*-oxide derivative were detected in the green propolis sample, alongside phenethylamine, methyltryptamine, and various tryptamine derivatives. These findings align with previous reports identifying DMT as a major compound in the bark of *M. tenuiflora* [31], while other tryptamine-type alkaloids and β-carbolines have been observed in its leaves and seeds [20]. The fragmentation patterns observed in the mass spectra, for tryptamine derivatives, provide additional evidence of their structural diversity and support the identification of these bioactive compounds in green propolis.

Previous studies demonstrating the broad-spectrum antibacterial properties of green propolis from the Caatinga biome, collected in Barauna, Rio Grande do Norte State. Specifically, it has shown inhibitory effect against important veterinary pathogens, including *Enterobacter aerogenes*, *Escherichia coli*, *Staphylococcus aureus*, and *Salmonella* spp. [32]. Fraction 14 displayed the most significant antimicrobial activity, especially with a MIC of 16.99 µg/mL against VRE, indicating it as a promising candidate for further study against drug-resistant bacteria. From this fraction, hispidulin and quercetin were isolated. In contrast, individual compounds exhibited minimum inhibitory concentrations (MICs) greater than 20 µg/mL for all tested strains. This indicates limited antimicrobial activity at the concentrations tested, suggesting that these flavonoids, while potentially beneficial for their other pharmacological properties (e.g., antioxidant effects), may not contribute significantly to the antimicrobial potency of propolis in isolation.

Studies by Bankova et al. [33] suggest that, in terms of antibacterial activity, no single component has shown greater efficacy than the total extract of propolis. Consequently, the fractionation process can isolate and concentrate bioactive compounds that are more effective in inhibiting microbial growth than the individual compounds by themselves. These findings align with previous research, which noted that, while flavonoids in propolis have multiple bioactivities, some of these effects often require synergistic interactions to achieve meaningful efficacy [16].

Overall, the results suggest that, while whole extracts and specific fractions of propolis exhibit antimicrobial effects against select pathogens, particularly resistant bacterial strains, the isolated flavonoids are less effective individually. Further research could explore the synergistic effects of these compounds when combined, as well as their potential applications in combating drug-resistant infections.

## 4. Materials and Methods

### 4.1. Propolis Collection and Extraction

The Brazilian green propolis from Caatinga was produced by Africanized honeybees (*Apis mellifera* L.) in Remanso, Bahia, Brazil, and collected in October 2022. Propolis (500 g) was frozen, grounded, and macerated with methanol–water, 10 (*w*/*v*) percentage, using a shaker incubator at 30 °C and 120 rpm, for 24 h; this was repeated twice. The combined extract was concentrated under vacuum using a Buchi rotary evaporator and lyophilized, yielding the crude extract (180 g).

### 4.2. General Experimental Procedures

TLC was performed on pre-coated SiO_2_ 60 F_254_ TLC plates (0.2 mm). The detection of the compounds used UV absorption (λ_max_ 254 and 366 nm) and, for derivatization, anisaldehyde reagent. NMR spectra of the isolated compounds were acquired to identify the chemical structures on a Bruker-Avance DRX 400 spectrometer (Bruker), operating at 400 MHz for ^1^H and 101 MHz for ^13^C NMR, respectively. Additionally, two-dimensional NMR experiments, including heteronuclear single quantum coherence (HSQC) and heteronuclear multiple bond correlation (HMBC), were conducted to further confirm the structural assignments (See Supplementary Material)The compounds were dissolved in deuterated methanol (CD_3_OD), acetone (CD_3_COCD_3_), chloroform (CDCl_3_), or DMSO (C_2_D_6_OS) according to their solubility. The NMR data were compared with these available in the literature to confirm the structure of the isolated compounds. The HR-ESI-MS analysis was carried out in an Agilent 1100 Series HPLC system (Agilent, Santa Clara, CA, USA) equipped with a Quaternary pump and an autosampler, hyphenated to a ToF-MS (Model #G6230B, Agilent Technologies, Santa Clara, CA, USA), equipped with electrospray ionization interface (ESI) source. All operations, data acquisition, and analysis were controlled using MassHunter Qualitative Analysis Software Ver. B.7.00. For the purification of the compounds, a preparative HPLC system (Agilent 1200 Series, Santa Clara, CA, USA) was employed. This system featured a G1361A binary pump, a G2260A autosampler, a G1315A diode array detector, and a G1364B fraction collector (Santa Clara, CA, USA). The acetonitrile, methanol, and formic acid were of HPLC certified grade (99.9% of purity, Thermo Fishcer Scientific, Fair Lawn, NJ, USA), and water was purified using a Milli-Q system (Millipore, Bedford, MA, USA) for the HR-ESI-MS analysis and QToF-MS-MS analysis. For other chromatographic processes, 99.8% purity laboratory-grade dichloromethane (DCM), chloroform, ethyl acetate (EtOAc), methanol (MeOH), and deionized water (DW) were purchased from Fisher Scientific (Thermo Fishcer Scientific, Fair Lawn, NJ, USA).

### 4.3. Chemical Characterization

The Caatinga green propolis extract (80 g) was submitted to a flash chromatography using the Isolera™ Prime (Biotage, Charlotte, USA) equipment. The stationary phase consisted of cartridges of silica gel Biotage^®^ SNAP Ultra C18 (350 g, HP-Sphere™, 25 µm). The mobile phase consisted of n-hexane (pump A), ethyl acetate (pump B), and methanol (pump C) in gradient mode (0–100 of A/B to 0–100 of B/C), with a flowrate of 100 mL/min, and a collection volume of 27 mL. Similar fractions were reunited according to their TLC profile, performed on pre-coated SiO_2_ 60 F254 TLC plates (0.2 mm). The detection of the compounds was monitored using UV absorption (λ_max_ 254 and 366 nm) and anisaldehyde reagent. A total of 35 fractions were obtained.

Fractions 8 (1 g) and 13 (850 mg), were fractionated by preparative RP-HPLC, with a C18 Phenomenex Luna C18 column (250 × 21.2 mm; 10 μm) using methanol (solvent B) and water (solvent A) in a gradient mode (30→40% of B in 4 min; 40→60% of B in 7 min; 60→80% of B in 10 min; hold 80% of B until 13 min; then 80→100% of B in 16 min). From F8 were isolated sakuranetin (187 mg) and penduletin (34 mg). From F13 was isolated kukulkanin B (10.6 mg).

Due to the complexity of F13, 3.22 g was subjected to flash chromatography using the Isolera™ Prime (Biotage), with a Biotage Sfar HC 100 g column, with ethyl acetate (pump B) and methanol (pump C) in gradient mode (30–100 of A/B to 0–100 of B/C), with a flowrate of 120 mL/min, and a collection volume of 27 mL. A total of 9 fractions were obtained. The subfraction F13.4 (162 mg) was submitted to a silica gel column (50 g) and eluted with dichloromethane and methanol (100:0 to 80:20), obtaining 7-*O*-methyleriodictyol (88 mg). Subfraction F13.5 (300 mg) was fractionated by Sephadex LH-20 (30 g), eluted with methanol. Compounds viscosine (16.5 mg), 6-methoxykaempferol (29.2), and kaempferol (3.9 mg) were isolated. Subfraction 13.9 was fractionated by Sephadex LH-20 (30 g), eluted with methanol, to obtain naringenin (33 mg).

Fraction 14 (3 g) was fractionated on a silica gel column (100 g) eluted with chloroform–methanol (100:0 to 80:20), reaching 14 subfractions. Subfraction F14.7 (580 mg) was submitted to a Sephadex LH-20 column (30 g) eluted with methanol, leading to the isolation of hispidulin (25 mg). Subfraction F14.12 (186.3 mg) was also fractionated on a Sephadex LH-20 column (30 g) eluted with methanol, reaching the isolation of quercetin (16.5 mg).

Fraction 15, 16, and 17 were joined (4.94 g) and submitted to a silica gel column (300 g), eluted with dichloromethane–methanol (100:0 to 60:40), obtaining 26 subfractions. Subfractions 15.2 and 3 were joined (300 mg) and fractionated on a silica gel column (9 g), eluted with dichloromethane–methanol (100:0 to 85:15). The separation led to the isolation of cirsimaritin (34.8 mg). Subfraction 15.17 was identified as quercetin 3-methyl ether (67.7 mg).

### 4.4. NMR and HRESI-MS Data (Two-Dimensional NMR See Supplementary Material)

Sakuranetin: Pale yellow amorphous powder; ^1^H NMR (400 MHz, CDCl_3_) δ 7.28 (d, *J* = 8.6 Hz, 2H, H-2′, H-6′), 6.81 (d, *J* = 8.5 Hz, 2H, H-3′, H-5′), 6.00 (s, 1H, H-6), 5.30 (dd, *J* = 13.1, 3.0 Hz, 1H, H-2), 3.77 (s, 3H, 7-OCH_3_), 3.09 (dd, *J* = 17.1, 13.0 Hz, 1H, H-3a), 2.68 (dd, *J* = 17.2, 3.0 Hz, 1H, H-3b); ^13^C NMR (101 MHz, CDCl_3_) δ 198.13 (C-4), 169.42 (C-7), 165.14 (C-5), 164.62 (C-8a), 159.02 (C-4′), 130.86 (C-1′), 129.04 (C-2′, C-6′), 116.32 (C-3′, C-5′), 104.01 (C-4a), 95.74 (C-6), 94.94 (C-8), 80.52 (C-2), 56.22 (7-OCH_3_), 43.97 (C-3); ^1^H and ^13^C NMR data as reported by Son et al. [28]; HRESIMS negative mode *m*/*z* 285.077 [M−H]^−^ (calcd. for C_16_H_14_O_5_, 286.070629).

Penduletin: Yellow amorphous powder; ^1^H NMR (400 MHz, CDCl_3_): δ 8.07 (d, *J* = 9.0 Hz, 2H, H-2′, H-6′), 7.02 (d, *J* = 9.1 Hz, 2H, H-3′, H-5′), 6.55 (s, 1H, H-8), 4.04 (s, 3H, 7-OCH_3_), 3.90 (s, 3H, 6-OCH_3_), 3.85 (s, 3H, 3-OCH_3_); ^13^C NMR (101 MHz, CDCl_3_) δ 179.26 (C-4), 161.80 (C-4′), 156.23 (C-7), 155.22 (C-2), 152.35 (C-8a), 151.96 (C-5), 138.51 (C-3), 130.26 (C-6), 122.84 (C-1′), 114.16 (C-3′, C-5′), 106.22 (C-4a), 93.28 (C-8), 60.97 (6-OCH_3_), 60.24 (7-OCH_3_), 55.53 (3-OCH_3_). ^1^H and ^13^C NMR data as reported by Son et al. [28]; HRESIMS negative mode *m*/*z* 343.084 [M−H]^−^ (calcd. for C_18_H_16_O_7_, 344.08958).

Kukulkanin B: Yellow amorphous powder; ^1^H NMR (400 MHz, MeOD) δ 7.78 (s, 1H, H-β), 7.76 (d, *J* = 9.1 Hz, 1H, H-6′), 7.62 (s, 1H, H-α), 7.61 (d, *J* = 8.8 Hz, 2H, H-2, H-6), 6.84 (d, *J* = 8.6 Hz, 2H, H-3, H-5), 6.48 (d, *J* = 8.9 Hz, 1H, H-5′), 3.85 (s, 3H, 3′-OCH_3_). ^13^C NMR (101 MHz, MeOD) δ 193.99 (C-9), 161.66 (C-4), 159.72 (C-4′), 158.54 (C-2′), 145.85 (C-β), 136.24 (C-3′), 131.89 (C-2, C-6), 127.74 (C-6′), 118.26 (C-α), 116.94 (C-3, C-5), 116.57 (C-1′), 109.01 (C-5′), 60.77 (3′-OCH_3_); ^1^H and ^13^C NMR data as reported by Dominguez et al. [30]; HRESIMS negative mode *m*/*z* 285.077 [M−H]^−^ (calcd. for C_15_H_11_O_4_, 286.07629).

7-*O*-Methyleriodictyol: Orange amorphous powder; ^1^H NMR (400 MHz, DMSO-*d*) δ 6.89 (s, 1H, H-2′), 6.75 (d, *J* = 1.2 Hz, 2H, H-5′, H-6′), 6.09 (d, *J* = 2.3 Hz, 1H, H-8), 6.07 (d, *J* = 2.3 Hz, 1H, H-6), 5.42 (dd, *J* = 12.6, 3.1 Hz, 1H, H-2), 3.78 (s, 3H, 7-OCH_3_), 3.23 (dd, *J* = 17.2, 12.6 Hz, 1H, H-3a), 2.72 (dd, *J* = 17.2, 3.1 Hz, 1H, H-3b); ^13^C NMR (101 MHz, DMSO-*d*) δ 196.98 (C-4), 167.46 (C-7), 163.25 (C-5), 162.87 (C-8a), 145.81 (C-4′), 145.25 (C-3′), 129.34 (C-1′), 118.05 (C-6′), 115.39 (C-5′), 114.42 (C-2′), 102.67 (C-4a), 94.63 (C-6), 93.83 (C-8), 78.71 (C-2), 55.92 (7-OCH_3_), 42.18 (C-3); ^1^H and ^13^C NMR data as reported by Ibrahim et al. [34]; HRESIMS negative mode *m*/*z* 301.064 [M−H]^−^ (calcd. for C_16_H_11_O_4_, 302.07119).

Viscosine: Yellow-brownish amorphous powder; ^1^H NMR (400 MHz, MeOD) δ 7.92 (d, *J* = 8.9 Hz, 2H, H-2′, H-6′), 6.89 (d, *J* = 8.9 Hz, 2H, H-3′, H-5′), 6.43 (s, 1H, H-8), 3.86 (s, 3H, 6-OCH_3_), 3.74 (s, 3H, 3-OCH_3_); ^13^C NMR (101 MHz, MeOD) δ 180.04 (C-4), 161.62 (C-4′), 159.83 (C-7), 157.92 (C-2), 153.78 (C-8a), 153.49 (C-5), 138.98 (C-3), 132.75 (C-6), 131.34 (C-2′, C-6′), 122.49 (C-1′), 116.55 (C-3′, C-5′), 105.94 (C-4a), 95.29 (C-8), 60.93 (6-OCH_3_), 60.53 (3-OCH_3_); ^1^H and ^13^C NMR data as reported by Son et al. [28]; HRESIMS negative mode *m*/*z* 329.067 [M−H]^−^ (calcd. for C_16_H_15_O_4_, 330.07392).

Kaempferol: Yellow amorphous powder; ^1^H NMR (400 MHz, MeOD) δ 8.06 (d, *J* = 8.9 Hz, 2H, H-2′, H-6′), 6.89 (d, *J* = 9.0 Hz, 2H, H-3′, H-5′), 6.37 (d, *J* = 2.0 Hz, 1H, H-8), 6.17 (d, *J* = 1.8 Hz, 1H, H-6); ^13^C NMR (101 MHz, MeOD) δ 177.31 (C-4), 165.59 (C-7), 162.45 (C-5), 160.49 (C-4′), 158.21 (C-8a), 148.00 (C-2), 130.65 (C-2′, C-6′), 123.72 (C-1′), 116.28 (C-3′, C-5′), 104.51 (C-4a), 99.28 (C-6), 94.48 (C-8); ^1^H and ^13^C NMR data as reported by To et al. [35]; HRESIMS negative mode *m*/*z* 285.035 [M−H]^−^ (calcd. for C_15_H_10_O_6_, 286.0477).

Naringenin: Yellow amorphous powder; ^1^H NMR (400 MHz, MeOD) δ 7.26 (d, *J* = 8.6 Hz, 2H, H-2′, H-6′), 6.80 (d, *J* = 8.6 Hz, 2H, H-3′, H-5′), 5.87 (s, 2H, H-6, H-8), 5.23 (dd, *J* = 13.1, 3.0 Hz, 1H, H-2), 3.03 (dd, *J* = 17.1, 13.1 Hz, 1H, H-3a), 2.62 (dd, *J* = 17.1, 3.0 Hz, 1H, H-3b); ^13^C NMR (101 MHz, MeOD) δ 197.65 (C-4), 168.19 (C-7), 164.71 (C-5), 158.79 (C-4′), 130.95 (C-1′), 128.98 (C-2′, C-6′), 116.27 (C-3′, C-5′), 103.26 (C-4a), 97.06 (C-6), 96.17 (C-8), 80.28 (C-2), 43.82 (C-3); ^1^H and ^13^C NMR data as reported by Cordenonsi et al. [36]; HRESIMS negative mode *m*/*z* 271.054 [M−H]^−^ (calcd. for C_15_H_12_O_5_, 272.06846).

6-Methoxykaempferol: Orange amorphous powder; ^1^H NMR (400 MHz, DMSO-*d*) δ 8.04 (d, *J* = 8.9 Hz, 2H, H-2′, H-6′), 6.92 (d, *J* = 9.0 Hz, 2H, H-3′, H-5′), 6.53 (s, 1H, H-8), 3.75 (s, 3H, 6-OCH_3_). ^13^C NMR (101 MHz, DMSO-*d*) δ 176.10 (C-4), 159.22 (C-4′), 157.42 (C-7), 151.71 (C-5), 151.44 (C-8a), 146.92 (C-3), 135.35 (C-2), 130.89 (C-6), 129.52 (C-2′, C-6′), 121.72 (C-1′), 115.44 (C-3′, C-5′), 103.34 (C-4a), 93.79 (C-8), 59.98 (6-OCH_3_); ^1^H and ^13^C NMR data as reported by Fuchino et al. [37]; HRESIMS negative mode *m*/*z* 315.044 [M−H]^−^ (calcd. for C_16_H_12_O_7_, 316.05826).

Quercetin: Yellow powder; ^1^H NMR (400 MHz, MeOD) δ 6.82 (d, *J* = 8.6 Hz, 1H, H-5′), 6.70 (d, *J* = 2.7 Hz, 1H, H-2′), 6.56 (dd, *J* = 8.6, 2.6 Hz, 1H, H-6′), 6.16 (s, 1H, H-6), 6.09 (d, *J* = 1.8 Hz, 1H, H-8); ^13^C NMR (101 MHz, MeOD) δ 179.69 (C-4), 165.19 (C-7), 162.61 (C-8a), 156.54 (C-5), 147.46 (C-4′), 145.32 (C-2), 145.05 (C-3′), 139.24 (C-3), 122.32 (C-1′), 116.57 (C-5′), 112.52 (C-6′), 109.19 (C-2′), 103.24 (C-4a), 100.39 (C-8), 94.98 (C-6); ^1^H and ^13^C NMR data as reported by Napolitano et al. [32]; HRESIMS negative mode *m*/*z* 301.043 [M−H]^−^ (calcd. for C_16_H_11_O_5_, 302.0426).

Hispidulin: Yellow amorphous powder; ^1^H NMR (400 MHz, DMSO-*d*) δ 7.88 (d, *J* = 8.9 Hz, 2H, H-2′, H-6′), 6.91 (d, *J* = 8.9 Hz, 2H, H-3′, H-5′), 6.73 (s, 1H, H-3), 6.56 (s, 1H, H-8), 3.75 (s, 4H, 6-OCH_3_); ^13^C NMR (101 MHz, DMSO-*d*) δ 182.14 (C-4), 163.82 (C-2), 161.19 (C-4′), 157.28 (C-7), 152.83 (C-5), 152.43 (C-8a), 131.36 (C-6), 128.45 (C-2′, C-6′), 121.28 (C-1′), 115.98 (C-3′, C-5′), 104.13 (C-4a), 102.41 (C-3), 94.27 (C-8), 59.98 (6-OCH_3_); ^1^H and ^13^C NMR data as reported by Barbosa et al. [38] HRESIMS negative mode *m*/*z* 315.051 [M−H]^−^ (calcd. for C_16_H_11_O_5_, 316.05826).

Cirsimaritin: Brownish amorphous powder; ^1^H NMR (400 MHz, DMSO-*d*) δ 7.87 (d, *J* = 8.4 Hz, 2H, H-2′, H-6′), 6.90 (d, *J* = 8.3 Hz, 2H, H-3′, H-5′), 6.77 (s, 1H), 6.72 (s, 1H, H-8), 3.87 (s, 3H, 6-OCH_3_), 3.72 (s, 3H, 7-OCH_3_); ^13^C NMR (101 MHz, DMSO-*d*) δ 182.11 (C-4), 163.96 (C-2), 161.27 (C-4′), 158.51 (C-7), 152.53 (C-8a), 152.09 (C-5), 131.81 (C-6), 128.40 (C-2′, C-6′), 121.10 (C-1′), 115.92 (C-3′, C-5′), 105.03 (C-4a), 102.61 (C-3), 91.35 (C-8), 59.97 (6-OCH_3_), 56.30 (7-OCH_3_); ^1^H and ^13^C NMR data as reported by Srivedavyasasri et al. [39]; HRESIMS negative mode *m*/*z* 313.072 [M−H]^−^ (calcd. for C_17_H_14_O_6_, 314.07902).

Quercetin 3 methyl ether: Yellow amorphous powder; ^1^H NMR (400 MHz, MeOD) δ 7.61 (d, *J* = 2.1 Hz, 1H, H-2′), 7.51 (dd, *J* = 8.5, 2.2 Hz, 1H, H-6′), 6.89 (d, *J* = 8.5 Hz, 1H, H-5′), 6.36 (d, *J* = 2.1 Hz, 1H, H-8), 6.17 (d, *J* = 2.0 Hz, 1H, H-6), 3.77 (s, 3H, 3-OCH_3_); ^13^C NMR (101 MHz, MeOD) δ 179.96 (C-4), 165.84 (C-7), 163.01 (C-5), 158.35 (C-8a), 157.95 (C-2), 149.90 (C-4′), 146.41 (C-3′), 139.50 (C-3), 122.91 (C-1′), 122.32 (C-6′), 116.46 (C-5′), 116.40 (C-2′), 105.83 (C-4a), 99.73 (C-6), 94.71 (C-8), 60.50 (3-OCH_3_); ^1^H and ^13^C NMR data as reported by Son et al. [28]; HRESIMS negative mode *m*/*z* 315.057 [M−H]^−^ (calcd. for C_16_H_12_O_7_, 316.05826).

### 4.5. Phytochemical Screening by LC-DAD-QToF-MS

#### 4.5.1. Sample Preparation

The extract was prepared in a 25 mg/mL concentration in HPLC-grade methanol, filtered using 0.45 µ PTFE filters before being subjected to LC-QToF-MS analysis.

#### 4.5.2. Instrumentation and Analytical Conditions

The phytochemical profile in Brazil propolis extract was determined using LC-DAD-QToF analysis. The analysis was performed using Agilent Series 1290 (Santa Clara, CA, USA) and the separation was achieved on an Poroshell 120 EC-C18 (Agilent Technologies, Santa Clara, CA, USA) (150 mm × 2.1 mm I.D., 2.7 µm particle size). The eluent system used a binary gradient consisted of water with 0.1% formic acid (A) and acetonitrile with 0.1% formic acid (B) at a flow rate of 0.23 mL/min using a following gradient elution: 0 min, 90% A/10% B isocratic for 2 min; 15 min, 30%B; in the next 20 min to 67%B; finally, to 100% B in the next 5 min. Each run was followed by a 5 min wash with 100% B and an equilibration period of 5 min with 90% A/10% B. Two microliters of sample were injected, and the column temperature was 35 °C. Phytochemical profiling was conducted by coupling the LC system to the quadrupole time-of-flight mass spectrometer equipped with an electrospray ionization interface (ESI). Nitrogen was used as the desolvation gas and set to 275 °C with a flow rate of 9 L/min. Other parameters used were nebulizer pressure, 30 psig, sheath gas temperature, 325 °C; sheath gas flow, 10 L/min; capillary voltage, 3000 V; and fragmentor voltage, 175 V. The mass analysis range was 50–1700 *m*/*z* and UV range 200–400 nm. Accurate mass measurements were obtained by means of reference ion correction using reference masses at *m*/*z* 121.0509 (protonated purine) and 922.0098 [protonated hexakis (1H, 1H, 3H-tetrafluoropropoxy) phosphazine or HP-921] in positive ion mode. Samples were analyzed in all-ion MS-MS mode, where experiment 1 was carried out with collision energy of zero and experiment two with a fixed collision energy of 45 eV. The precise molecular mass and molecular formula were processed with MassHunter Qualitative Analysis Software Ver. B.7.00. A subset of compounds was identified through comparison of their MS data and retention times to isolated standards, whereas others were tentatively identified and annotated utilizing mass spectral data from the published literature.

### 4.6. Antimicrobial Assessment

The antimicrobial activities of the crude extract, fractions, and compounds were tested against *Candida albicans* ATCC 90028, *Aspergillus fumigatus* ATCC 204305, *Cryptococcus neoformans* ATCC 90113, methicillin-resistant *Staphylococcus aureus* (MRS) ATCC 1708, *Escherichia coli* ATCC 2452, *Pseudomonas aeruginosa* ATCC BAA-2018, *Klebsiella pneumoniae* ATCC 2146, and *Enterococcus faecium* (VRE) ATCC 700221. The strains used in this study were obtained from the American Type Culture Collection (ATCC, Manassas, VA). Antimicrobial susceptibility testing was carried out using an adapted version of the CLSI method [40]. Crude extract was evaluated at concentrations of 8–200 µg/mL, while fractions and pure compounds were tested at 0.8–20 µg/mL. The growth of inocula and final reader conditions were based on previous stablished protocols [41]. For each assay, appropriate drug controls were included for both bacterial and fungal strains. The assay was performed in duplicate. IC_50_ values were determined using XLfit 4.2 software (IDBS, Alameda, CA, USA) with the fit model 201.

## 5. Conclusions

The green propolis from the Brazilian Caatinga biome is characterized by a rich and diverse chemical composition, with high concentrations of flavonoids. The biological profiles of Caatinga green propolis underscore moderate antimicrobial potential. Our study identified a rich presence of flavonoids and phenolic acids, revealing that the chemical composition of green propolis from the Caatinga biome is both complex and distinctive. While individual flavonoids exhibited limited antimicrobial activity, the fractions derived from propolis demonstrated significant inhibitory effects, particularly against drug-resistant pathogens like MRSA and VRE. These results suggest that the effectiveness of green propolis may be attributed to the synergistic interactions between its constituents, rather than the activity of single compounds alone. Future studies should explore the synergistic mechanisms underlying these effects and evaluate the therapeutic potential of propolis in managing infections caused by resistant bacteria.

## Figures and Tables

**Table 1:** Tentative identification of compounds in Brazilian green propolis of Caatinga extract using LC-Q-ToF in positive and negative ionization mode.

#	RT (min)	Compound Name	Molecular Formula	Mass	[M+H]^+^	Fragment Ions (Positive Mode)	[M−-H]^−^	Fragment Ions (Negative mode)
**Reference compounds (1–14)**								
1	1.65	Quinic acid [19]	C_7_H_12_O_6_	192.0634	193.0710 (193.0707) *	-	191.0558 (191.0561)	173.0455 [M−-H−-H_2_O]^−^, 155.0349 [M−H−2H_2_O]^−^
2	7.10	Caffeic acid [19]	C_9_H_8_O_4_	180.0423	181.0498 (181.0495)	-	179.0349 (179.0350)	161.0027 [M−H−H_2_O]^−^, 135.0452 [M−H−CO_2_]^−^
3	18.00	Quercetin	C_15_H_10_O_7_	302.0427	303.0482 (303.0499)	285.0544 [M+H−CH_3_]^+^, 257.0445 [M+H−H_2_O−CO]^+^, 229.0478 [M+H−H_2_O−2CO]^+^, 195.0295 [M+H−C_6_H_4_O_2_]^+^, 153.0184 [M+H− C_7_H_6_O_2_−CO]^+^, 109.0285 [M+H−C_7_H_6_O_2_−CO−CO_2_]^+^	301.0355 (301.0354)	257.0460 [M−H−CO_2_]^−^, 193.0152 [M−H−C_6_H_4_O_2_]^−^, 151.0036 [M−H−C_7_H_6_O_2_−CO]^−^, 121.0292 [M−H−C_8_H_4_O_5_]^−^, 107.0139 [M−H-C_7_H_6_O_2_−CO−CO_2_]^−^
4	18.65	Quercetin-3-methyl ether	C_16_H_12_O_7_	316.0583	317.0653 (317.0656)	302.0403 [M+H−CH_3_]^+^, 273.0378 [M+H−CH_3_−(H+CO)]^+^, 153.0174 [M+H−C_9_H_8_O_3_]^+^, 109.0272 [M+H−C_10_H_8_O_5_]^+^	315.0511 (315.0510)	300.0273 [M−H−CH_3_]^−^, 271.0247 [M−H−CH_3_−(H+CO)]^−^, 151.0043 [M−H−C_9_H_8_O_3_]^−^, 107.0150 [M−H−C_10_H_8_O_5_]^−^
5	19.98	Naringenin	C_15_H_12_O_5_	272.0685	273.0756 (273.0757)	269.0472 [M+H−H_2_O]^+^, 241.0526 [M+H−H_2_O−CO]^+^, 153.0176 [M+H−C_8_H_8_O]^+^, 121.0647 [M+H−C_7_H_4_O_4_]^+^	271.0612 (271.0612)	151.0037 [M−H−C_8_H_8_O]^−^, 119.0498 [M−H−C_7_H_4_O_4_]^−^
6	20.35	Hispidulin	C_16_H_12_O_6_	300.0634	301.0701 (301.0707)	286.0480 [M+H−CH_3_]^+^, 168.0063 [M+H−CH_3_−C_8_H_6_O]^+^	299.0554 (299.0561)	284.0325 [M−H−CH_3_]^−^, 117.0348 [M−H−C_8_H_6_O_5_]^−^
7	20.58	Kaempferol	C_15_H_10_O_6_	286.0477	287.0567 (287.0550)	241.0412 [M+H−H_2_O−CO]^+^, 231.0475 [M+H-H_2_O−2CO]^+^, 153.0138 [M+H−C_7_H_6_O−CO]^+^, 147.0408 [M+H−2CO−C_4_H_4_O_2_]^+^, 107.0478 [M+H−C_8_H_4_O_5_]^+^	285.0402 (285.0405)	241.0514 [M−H−CO_2_]^−^, 257.0468 [M−H−CO]^−^, 193.0098 [M−H−C_6_H_4_O]^−^, 178.9986 [M−H−C_7_H_6_O]^−^, 105.0348 [M−H−C_8_H_4_O_5_]^−^, 153.0058 [M−H−C_7_H_6_O−CO]^−^, 107.0143 [M−H−C_7_H_6_O−CO−CO_2_]^−^
8	20.72	6-Methoxykaempferol	C_16_H_12_O_7_	316.0583	317.0645 (317.0656)	302.0395 [M+H−CH_3_]^+^, 273.0387 [M+H−CH_3_−(H+CO]^+^, 245.0576 [M+H−CH_3_−(H+CO)−CO]^+^	315.0506 (315.0510)	300.0269 [M−H−CH_3_]^−^, 271.0248 [M−H−CH_3_−(H+CO)]^−^, 243.0299 [M−H−CH_3_−(H+CO)−CO]^−^
9	21.84	Viscosine	C_17_H_14_O_7_	330.0740	331.0819 (331.0812)	316.0575 [M+H−CH_3_]^+^, 301.0383 [M+H−2CH_3_]^+^, 273.0385 [M+H−2CH_3_−CO]^+^, 211.0431 [M+H−C_7_H_4_O_2_]^+^, 123.0440 [M+H−C_10_H_8_O_5_]^+^	329.0666 (329.0667)	314.0427 [M−H−CH_3_]^−^, 299.0210 [M−H−2CH_3_]^−^, 271.0245 [M−H−2CH_3_−CO]^−^, 259.0612 [M−H−C_2_H_2_O−CO]^−^, 244.0346 [M−H−C_2_H_2_O−CO−CH_3_]^−^, 182.0221 [M−H−C_9_H_7_O_2_]^−^, 209.0456 [M−H−C_7_H_4_O_2_]^−^, 121.0295 [M−H−C_10_H_8_O_5_]^−^
10	22.75	Kukulkanin B	C_16_H_14_O_5_	286.0841	287.0919 (287.0914)	121.0605 [M+H−C_8_H_6_O_4_]^+^, 167.0369 [M+H−C_8_H_8_O]^+^, 152.0132 [M+H−C_9_H_11_O]^+^	285.0775 (285.0768)	119.0506 [M−H−C_8_H_6_O_4_]^−^, 165.0201 [M−H−C_8_H_8_O]^−^
11	23.20	7-*O-*Methyleriodictyol	C_16_H_14_O_6_	302.0790	303.0866 (303.0863)	167.0331 [M+H−C_8_H_8_O_2_]^+^, 137.0597 [M+H−C_8_H_6_O_4_]^+^	301.0720 (301.0718)	165.0193 [M−H−C_8_H_8_O_2_]^−^, 135.0450 [M−H−C_8_H_6_O_4_]^−^
12	23.82	Cirsimaritin	C_17_H_14_O_6_	314.0790	315.0865 (315.0863)	300.0632 [M+H−CH_3_]^+^, 285.0393 [M+H−2CH_3_]^+^, 136.0162 [M+H−C_10_H_11_O_3_]^+^, 119.0492 [M+H−C_9_H_8_O_5_]^+^, 108.0212 [M+H−C_11_H_11_O_4_]^+^	313.0719 (313.0718)	298.0469 [M−H−CH_3_]^−^, 283.0246 [M−H−2CH_3_]^−^, 117.0347 [M−H−C_9_H_8_O_5_]^−^
13	26.23	Sakuranetin	C_16_H_14_O_5_	286.0841	287.0911 (287.0914)	167.0328 [M+H−C_8_H_8_O]^+^	285.0770 (285.0768)	270.0533 [M−H−CH_3_]^−^, 165.0186 [M−H−C_8_H_8_O]^−^, 119.0496 [M−H−CH_3_−C_7_H_3_O_4_ ]^−^
14	28.62	Penduletin	C_18_H_16_O_7_	344.0896	345.0957 (345.0969)	330.0700 [M+H−CH_3_]^+^, 315.0474 [M+H−2CH_3_]^+^, 300.0264 [M+H−3CH_3_]^+^, 287.0542 [M+H−C_3_H_6_O]^+^	343.0817 (343.0823)	328.0581 [M−H−CH_3_]^−^, 313.0340 [M−H−2CH_3_]^−^, 298.0135 [M−H−3CH_3_]^−^, 270.0172 [M−H−3CH_3_−CO]^−^, 186.0318 [M−H−C_7_H_9_O_4_]^−^
**Alkaloids (15–25)**								
15	2.80	Dimethyltryptamine *N*-oxide	C_12_H_16_N_2_O	204.1263	205.1325 (205.1335)	191.1170 [M+H−CH_2_]^+^, 173.1090 [M+H−CH_2_−H_2_O]^+^, 130.0665 [C_9_H_7_N+H]^+^, 117.0587 [C_9_H_7_+H−NH]^+^, 91.0555, 77.0406	-	-
16	3.66	Phenethylamine	C_8_H_11_N	121.0891	122.0954 (122.0964)	105.0712 [M+H−NH_3_]^+^, 77.0405 [M+H−NH_3_−C_2_H_4_]^+^	-	-
17	4.17	Methylphenethylamine	C_9_H_13_N	135.1048	136.1127 (136.1121)	105.0726, 77.0403	-	-
18	5.32	Tryptamine derivative	C_14_H_18_N_2_O	230.1419	231.1484 (231.1492)	160.0722, 142.0615, 132.0628, 115.0499, 91.0508, 77.0349, 65.0349, 51.0199	-	-
19	5.81	Tryptamine [20]	C_10_H_12_N_2_	160.1000	161.1084 (161.1073)	144.0815, 142.0652, 127.0540, 115.0535, 91.0538, 77.0381, 65.0384, 51.0216	-	-
20	6.50	Methyltryptamine [20]	C_11_H_14_N_2_	174.1157	175.1216 (175.1230)	144.0815 [M+H−CH_3_−NH_2_]^+^, 142.0665 [M+H−CH_3_−NH_2_−H_2_]^+^, 127.0543 [M+H−CH_3_−NH_2_−H_2_−NH]^+^, 115.0544 [C_9_H_6_+H]^+^, 91.0549 [C_7_H_6_+H]^+^, 77.0391 [C_6_H_4_+H]^+^, 65.0387 [C_5_H_4_]^+^, 51.0235 [C_4_H_2_+H]^+^	-	-
21	7.00	Dimethyltryptamine [21,22]	C_12_H_16_N_2_	188.1313	189.1391 (189.1386)	175.1251, 144.0820, 142.0661, 127.0544, 115.0550, 91.0547, 77.0389, 51.0226	-	-
22	7.80	Tryptamine derivative	C_11_H_12_N_2_	172.1000	173.1082 (173.1073)	144.0820, 142.0630, 127.0550, 115.0539, 91.0534, 77.0383, 51.0237	-	-
23	8.66	Methyl Tetrahydro-β-carboline [20,22]	C_12_H_14_N_2_	186.1157	187.1246 (187.1230)	144.0812, 115.0521, 91.0547, 77.0381, 51.0230	-	-
24	9.29							
25	10.04	Ethyl Tetrahydro-β-carboline	C_13_H_16_N_2_	200.1313	201.1399 (201.1386)	175.1230, 158.0958, 144.0795, 142.0631, 115.0531, 91.0534, 77.0383, 51.0211	-	-
**Phenolic acid derivatives (26–33)**								
26	2.22	Gallic acid [23]	C_7_H_6_O_5_	170.0215	-	-	169.0143 (169.0142)	125.0241 [M−H−CO_2_]^−^
27	2.89	Gallic acid derivatives	C_19_H_20_O_13_	456.0904	457.0982 (457.0977)	153.0184 [C_7_H_4_O_4_+H]^+^	455.0839 (455.0831)	303.0707 [M−H−C_7_H_4_O_4_]^−^, 285.0611 [M−H−C_7_H_4_O_4_−H_2_O]^−^, 169.0142 [gallic acid−H]^−^, 125.0239 [gallic acid−H−CO_2_]^−^
28	4.00							
29	4.95	Gallic acid derivative (4-*O*-galloylarbutin)	C_19_H_20_O_11_	424.1006	-	-	423.0930 (423.0933)	313.0568 [M−H−C_6_H_6_O_2_]-, 211.0241 [C_9_H_8_O_6_−H]-, 169.0138 [gallic acid−H]^−^, 125.0247 [gallic acid−H−CO_2_]^−^
30	5.90	Methyl gallate	C_8_H_8_O_5_	184.0372	-	-	183.0299 (183.0299)	169.0126, 124.0165, 78.0120
31	7.52	Gallic acid derivative	C_19_H_20_O_12_	440.0955	-	-	439.0871 (439.0882)	285.0584 [M−H−C_7_H_6_O_4_]-, 169.0145, 125.0247
32	8.30	Gallic acid derivative	C_26_H_24_O_17_	608.1013	609.1104 (609.1086)	153.0187 [C_7_H_4_O_4_+H]^+^	607.0947 (607.0941)	455.0818 [M−H−C_7_H_4_O_4_]^−^, 437.0719 [M−H−C_7_H_4_O_4_−H_2_O]^−^, 303.0698 [M−H−2C_7_H_4_O_4_]^−^, 285.0608 [M−H−2C_7_H_4_O_4_−H_2_O]^−^, 267.0509 [M−H−2C_7_H_4_O_4_−2H_2_O]^−^, 169.0138 [gallic acid−H]^−^, 125.0250 [gallic acid−H−CO_2_]^−^
33	9.87	Ethyl gallate	C_9_H_10_O_5_	198.0528	-	-	197.0466 (197.0455)	169.0129, 124.0161, 78.0119
**Flavonols (Flavonoids) (34–56)**								
34	11.00	Isoquercetin/Hyperoside	C_21_H_20_O_12_	464.0955	465.1033 (465.1028)	303.0499 [M+H−Hexose]^+^, 85.0272	463.0876 (483.0882)	300.0272 [M−H−Hexose]^−^
35	11.17							
36	11.44							
37	14.68							
38	12.00	Quercetin-pentoside	C_20_H_18_O_11_	434.0849	435.0918 (435.0922)	303.0505 [M+H−Pentose]^+^	433.0773 (433.0776)	300.0290 [M−H−Pentose]^−^
39	12.32							
40	12.71							
41	12.35	Kaempferol-glucoside	C_21_H_20_O_11_	448.1006	449.1092 (449.1078)	287.0539 [M+H−Rhamnose]^+^	447.0923 (447.0933)	285.0391
42	12.86							
43	12.61	Isorhamnetin glucoside	C_22_H_22_O_12_	478.1111	479.1186 (479.1184)	317.0660, 302.0404, 153.0181	477.1027 (477.1038)	315.0494, 299.0189
44	13.14							
45	13.00	Quercetin-rhamnoside	C_21_H_20_O_11_	448.1006	449.1094 (449.1078)	303.0504	447.0926 (447.0933)	300.0261, 301.0333
46	14.05							
47	14.56							
48	15.07							
49	17.55	Herbacetin	C_15_H_10_O_7_	302.0427	303.0485 (303.0499)	153.0190	301.0356 (301.0354)	273.0405, 178.9999, 151.0042,
50	18.30	Laricitrin [24]	C_16_H_12_O_8_	332.0532	333.0600 (333.0605)	318.0347 [M+H−CH_3_]^+^, 210.0170 [M+H−C_7_H_7_O_2_]^+^, 183.0275 [M+H−C_8_H_6_O_3_]^+^	331.0467 (331.0459)	316.0229 [M−H−CH_3_]^−^, 223.0258 [M−H−C_6_H_4_O_2_]^−^, 208.0024 [M−H−C_7_H_7_O_2_]^−^, 181.0144 [M−H−C_8_H_6_O_3_]^−^
51	18.85	Axillarin	C_17_H_14_O_8_	346.0689	347.0759 (347.0761)	313.0388, 289.0354 [M+H−2CH_3_−CO]^+^, 269.0499, 153.0172 [M+H−C_10_H_10_O_4_]^+^	345.0613 (345.0616)	330.0375 [M−H−CH_3_]^−^, 315.0169 [M−H−2CH_3_]^−^, 287.0194 [M−H−2CH_3_−CO]^−^
52	20.94	Trihydroxy dimethoxy flavone isomers	C_17_H_14_O_7_	330.0740	331.0810 (331.0812)	316.0567 [M+H−CH_3_]^+^, 301.0343 [M+H−2CH_3_]^+^, 273.0378 [M+H−2CH_3_−CO]^+^, 245.0456 [M+H−2C_2_H_2_−CO]^+^, 153.0179 [M+H−C_10_H_10_O_3_]^+^	329.0664 (329.0667)	314.0432 [M−H−CH_3_]^−^, 299.0206 [M−H−2CH_3_]^−^, 271.0242 [M−H−2CH_3_−CO]^−^
53	22.00							
54	24.70							
55	27.11	Acetyl-pinobanksin [19]	C_17_H_14_O_6_	314.0790	315.0870 (315.0863)	300.0654 [M+H−CH_3_]^+^, 285.0411 [M+H−2CH_3_]^+^, 272.0689 [M+H−CH_3_−CO]^+^, 257.0456 [M+H−2CH_3_−CO]^+^, 243.0659 [M+H−CH_3_−CO−CHO]^+^, 201.0557 [M+H−CH_3_−CO−CHO−C_2_H_2_O]^+^, 167.0339 [C_8_H_6_O_4_+H]^+^	313.0721 (313.0718)	298.0466 [M−H−CH_3_]^−^, 283.0243 [M−H−2CH_3_]^−^, 255.0285 [M−H−2CH_3_−CO]^−^
56	27.97							
**Flavones (Flavonoids) (57–64)**								
57	17.40	Luteolin	C_15_H_10_O_6_	286.0047	287.0550 (287.0550)	153.0177 [M+H−C_8_H_6_O_2_]^+^, 135.0440 [M+H−C_7_H_4_O_4_]^+^	285.0409 (285.0405)	151.0037 [M−H−C_8_H_6_O_2_]^−^, 133.0298 [M−H−C_7_H_4_O_4_]^−^, 107.0136
58	19.95	Apigenin	C_15_H_10_O_5_	270.0528	271.0609 (271.0601)	153.0198, 119.0488, 91.0547	269.0453 (269.0455)	151.0025, 117.0345
59	21.55	Luteolin methyl-ether [19]	C_16_H_12_O_6_	300.0634	301.0702 (301.0707)	286.0447 [M+H−CH_3_]^+^, 258.0527 [M+H−CH_3_−CO]^+^, 229.0860 [M+H−CO_2_−CO]^+^, 153.0183 [M+H−C_8_H_6_O_2_]^+^, 135.0449 [M+H−C_7_H_4_O_4_]^+^	299.0563 (299.0561)	284.0324 [M−H−CH_3_]^−^, 256.0527 [M−H−CH_3_−CO]^−^, 255.0663 [M−H−CO_2_]^−^, 211.0765 [M−H−2CO_2_]^−^, 151.0036 [M−H−C_8_H_6_O_2_]^−^, 133.0292 [M−H−C_7_H_4_O_4_]^−^
60	24.40	Dihydroxy-trimethoxy flavones	C_18_H_16_O_7_	344.0896	345.0965 (345.0969)	329.0636 [M+H−CH_4_]^+^, 311.0554 [M+H−CH_4_−H_2_O]^+^, 287.0575 [M+H−CH_4_−C_2_H_2_O]^+^, 269.0459 [M+H−CH_4_−C_2_H_2_O−H_2_O]^+^, 267.0567 [M+H−CH_4_−H_2_O−CO_2_]^+^, 255.0641 [M+H−CH_4_−H_2_O−CO_2_−C]^+^, 241.0507 [M+H−CH_4_−H_2_O−CO_2_−C−CH_2_]^+^, 213.0534 [M+H−CH_4_−H_2_O−CO_2_−C−CH_2_−CO]^+^, 187.0372 [M+H−CH_4_−H_2_O−CO_2_−C−CH_2_−CO−C_2_H_2_]^+^, 165.0167 [C_8_H_4_O_4_+H]^+^	343.0830 (343.0823)	298.0115 [M−H−3CH_3_]^−^, 270.0165 [M−H−3CH_3_−CO]^−^, 242.0210 [M−H−3CH_3_−2CO]^−^, 214.0265 [M−H−3CH_3_−3CO]^−^, 186.0323 [M−H−3CH_3_−4CO]^−^
61	25.24							
62	28.34		C_18_H_16_O_7_	344.0896	345.0965 (345.0969)	330.0735 [M+H−CH_3_]^+^, 315.0492 [M+H−2CH_3_]^+^, 287.0604 [M+H−2CH_3_−CO]^+^, 259.0618 [M+H−2CH_3_−2CO]^+^, 231.0668 [M+H−2CH_3_−3CO]^+^, 216.0423 [M+H−3CH_3_−3CO]^+^, 203.0724 [M+H−3CH_3_−4CO]^+^, 167.0353 [C_8_H_6_O_4_+H]^+^	343.0830 (343.0823)	328.0548 [M−H−CH_3_]^−^, 313.0306 [M−H−2CH_3_]^−^, 299.0542 [M−H−2CH_2_O]^−^, 284.0312 [M−H−2CH_2_O−CH_3_]^−^
63	25.80	Dihydroxy-tetramethoxyflavone	C_19_H_18_O_8_	374.1002	375.1075 (375.1074)	359.0640 [M+H−CH_4_]^+^, 345.0640 [M+H−CH_2_−CH_4_]^+^, 329.0640 [M+H−CH_4_−CH_2_O]^+^, 317.0687 [M+H−CH_4_−C_2_H_2_O]^+^, 299.0583 [M+H−CH_4_−C_2_H_2_O−H_2_O]^+^, 271.0619 [M+H−CH_4_−C_2_H_2_O−H_2_O−CO]^+^, 243.0679 [M+H−CH_4_−C_2_H_2_O−H_2_O−2CO]^+^, 191.0727 [C_11_H_10_O+H]^+^, 165.0545 [C_11_H_10_O+H−C_2_H_2_]^+^	373.0922 (373.0929)	-
64	26.50	Acacetin	C_16_H_12_O_5_	284.0685	285.0749 (285.0757)	242.0520 [M+H−CH_3_−CO]^+^, 153.0149 [M+H−C_9_H_8_O]^+^	283.0615 (283.0612)	268.0378 [M−H−CH_3_]^−^
**Flavan-3-ol (Flavonoid) (65)**								
65	8.05	(Epi)gallocatechin gallate [21]	C_22_H_18_O_11_	458.0849	459.0913 (459.0922)	289.0694 [M+H−C_7_H_6_O_5_]^+^, 151.0389, 139.0387	457.0772 (457.0776)	169.0132, 125.0247
**Flavanones (Flavonoids) (66–67)**								
66	17.10	Eriodictyol [19]	C_15_H_12_O_6_	288.0634	289.0706 (289.0707)	153.0191 [M+H−C_8_H_8_O_2_]^+^, 135.0423 [M+H−C_7_H_4_O_2_]^+^	287.0563 (287.0561)	151.0042 [M−H−C_8_H_8_O_2_]^−^, 135.0456 [M−H−C_7_H_4_O_2_]^−^
67	22.33	3-Acetoxyhesperitin	C_18_H_16_O_8_	360.0845	361.0933 (361.0918)	303.0511, 151.0376	359.0765 (359.0772)	301.0341
**Pterocarpan (Isoflavonoid) (68)**								
68	23.46	Melilotocarpan D [25]	C_17_H_16_O_6_	316.0947	317.1017 (317.1020)	197.0442 [C_9_H_8_O_5_+H]^+^, 182.0198 [C_9_H_8_O_5_+H−CH_3_]^+^, 164.0087 [C_9_H_8_O_5_+H−CH_3_−H_2_O]^+^, 153.0183 [C_9_H_8_O_5_+H−CH_3_−CHO]^+^, 136.0139 [C_9_H_8_O_5_+H−CH_3_−CHO−OH]^+^, 108.0182 [C_9_H_8_O_5_+H−CH_3_−CHO−OH−CO]^+^	315.0870 (315.0874)	-
**Isoflavone (Flavonoid) (69)**								
69	26.84	Dihydroxy-dimethoxyisoflavone	C_17_H_14_O_6_	314.0790	315.0869 (315.0863)	301.0739 [M+H−CH_2_]^+^, 285.0780 [M+H−CH_2_O]^+^, 257.0455 [M+H−CH_2_O−C_2_H_4_]^+^, 229.0514 [M+H−CH_2_O−C_2_H_4_−CO]^+^	313.0717 (313.0718)	299.0559 [M−H−CH_2_]^−^, 283.0624 [M−H−CH_2_O]^−^

Note: ‘*’ indicates theoretical accurate mass: CH_3_: 15.0235 Da; CO: 27.9949 Da; CO_2_:43.9898 Da; CH_2_O:30.0106 Da; H_2_O:18.0106 Da; CH_4_: 16.0313; C_2_H_4_: 28.0313; NH: 15.0109; NH_2_: 16.0182; NH_3_: 17.0265; Rhamnose: 146.0579; Pentose: 132.0423; Hexose: 162.0528.

**Table 2:** Antimicrobial activity of Brazilian green propolis crude extract, fractions, and isolated compounds.

	IC_50_ (μg/mL)		
Extract/Compound	*C. neoformans*	Methicillin-Resistant *S. aureus*	*E. faecium* (VRE)
Propolis extract	192.50	148.44	120.98
Fraction 8	47.86	142.34	101.89
Fraction 14	164.50	181.82	16.99
Sakuranetin	NA	NA	NA
Penduletin	NA	NA	NA
7-*O*-Methyleriodictyol	NA	NA	NA
Naringenin	NA	NA	NA
Quercetin	NA	NA	NA
Hispidulin	NA	NA	NA
Cirsimaritin	NA	NA	NA
3-Methyl quercetin	NA	NA	NA
6-Methoxy kaempferol	NA	NA	NA
Amphotericin B	0.46	-	NT
Meropenem	-	7.03	NA

VRE = Vancomycin-resistant Enterococci. NA = not active up to 200 µg/mL for extracts/fraction and 20 µg/mL for pure compounds. NT = not tested.

## Data Availability

Data are contained within the article.

## Supplementary Materials

The following supporting information can be downloaded at: https://www.mdpi.com/article/10.3390/plants13243576/s1, Figure S1: ^1^H and ^13^C NMR (MeOD; 500 MHz) of sakuranetin; Figure S2: HSQC and HMBC NMR (MeOD; 400 MHz) of sakuranetin; Figure S3: ^1^H and ^13^C NMR (MeOD; 400 MHz) of penduletin; Figure S4: HSQC and HMBC NMR (CDCl_3_; 400 MHz) of penduletin; Figure S5: ^1^H and ^13^C NMR (MeOD; 400 MHz) of kukulkanin B; Figure S6: HSQC and HMBC NMR (MeOD; 400 MHz) of kukulkanin B; Figure S7: ^1^H and ^13^C NMR (DMSO-*d*; 400 MHz) of 7-*O*-methyleriodictyol; Figure S8: HSQC and HMBC NMR (DMSO-*d*; 400 MHz) of 7-*O*-methyleriodictyol; Figure S9: ^1^H and ^13^C NMR (CD_3_OD; 400 MHz) of viscosine; Figure S10: HSQC and HMBC NMR (CD_3_OD; 400 MHz) of viscosine; Figure S11: ^1^H and ^13^C NMR (MeOD; 400 M Hz) of kaempferol; Figure S12: HSQC and HMBC NMR (MeOD; 400 MHz) of kaempferol; Figure S13: ^1^H and ^13^C NMR (MeOD; 400 MHz) of naringenin; Figure S14: HSQC and HMBC NMR (MeOD; 400 MHz) of naringenin; Figure S15: ^1^H and ^13^C NMR (DMSO-*d*; 400 MHz) of 6-methoxykaempferol; Figure S16: HSQC and HMBC NMR (DMSO-*d*; 400 MHz) of 6-methoxykaempferol; Figure S17: ^1^H and ^13^C NMR (MeOD; 400 MHz) of quercetin; Figure S18: HSQC and HMBC NMR (MeOD; 400 MHz) of quercetin; Figure S19: ^1^H and ^13^C NMR (DMSO-*d*; 400 MHz) of hispidulin; Figure S20: HSQC and HMBC NMR (DMSO-*d*; 400 MHz) of hispidulin; Figure S21: ^1^H and ^13^C NMR (MeOD; 400 MHz) of cirsimaritin; Figure S22: HSQC and HMBC NMR (MeOD; 400 MHz) of cirsimaritin; Figure S23: ^1^H and ^13^C NMR (MeOD; 400 MHz) of quercetin 3-methyl ether; Figure S24: HSQC and HMBC NMR (MeOD; 400 MHz) of quercetin 3-methyl ether.
