# Supplementary material for: Chemical Characterization and Antimicrobial Activity of Green Propolis from the Brazilian Caatinga Biome
Source: Plants (Basel). 2024 Dec 21;13(24):3576. doi: 10.3390/plants13243576 (PMC11677851; doi:10.3390/plants13243576)
Supplement: Supplementary file 1 [file plants-13-03576-s001.zip › plants-3357286-supplementary.pdf]

Supplementary Material

Chemical Characterization and Antimicrobial Activity of green propolis from the Brazilian Caatinga biome

Jennyfer A. Aldana-Mejía [1], Victor Pena Ribeiro [2], Kumar Katragunta [1], Bharathi Avula [1], Kiran Kumar Tatapudi [1], Jairo Kenupp Bastos [3], Ikhlas A. Khan [1,4], Kumudini Meepagala [2] and Samir A. Ross [1,3,*].

[1] National Center for Natural Products Research, School of Pharmacy, University of Mississippi, University, MS 38677 USA; jaaldana@olemiss.edu (J.A.A.-M.); kkatragu@olemiss.edu (K.K.); bavula@olemiss.edu (B.A.); kktatapu@olemiss.edu (K.K.T.); ikhan@olemiss.edu (I.A.K.)

[2] Agricultural Research Service, Natural Products Utilization Research Unit, U.S. Department of Agriculture, University, MS, 38677, USA; victor.ribeiro@usda.gov (V.P.R.); kumudini.meepagala@usda.gov (K.M.)

[3] School of Pharmaceutical Sciences of Ribeirão Preto, University of São Paulo, Av. do Café, Ribeirão Preto, 14040-930, Brazil; jkbastos@fcfrp.usp.br

[4] Division of Pharmacognosy, Department of BioMolecular Sciences, School of Pharmacy, University of Mississippi, University, MS 38677 USA

* Correspondence: sross@olemiss.edu

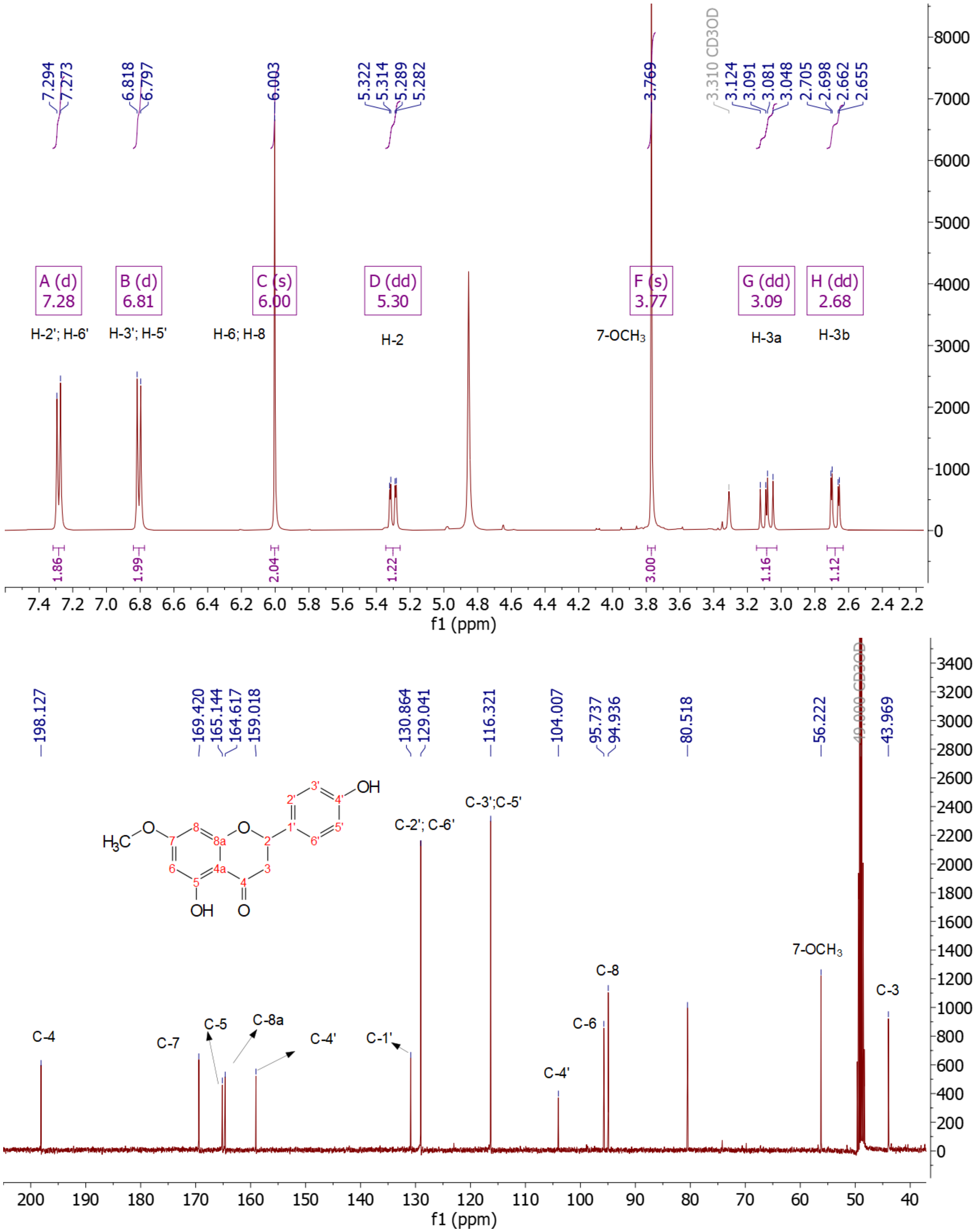

Figure S 1. ^{1}H and ^{13}C NMR (MeOD; 400 MHz) of sakuranetin.

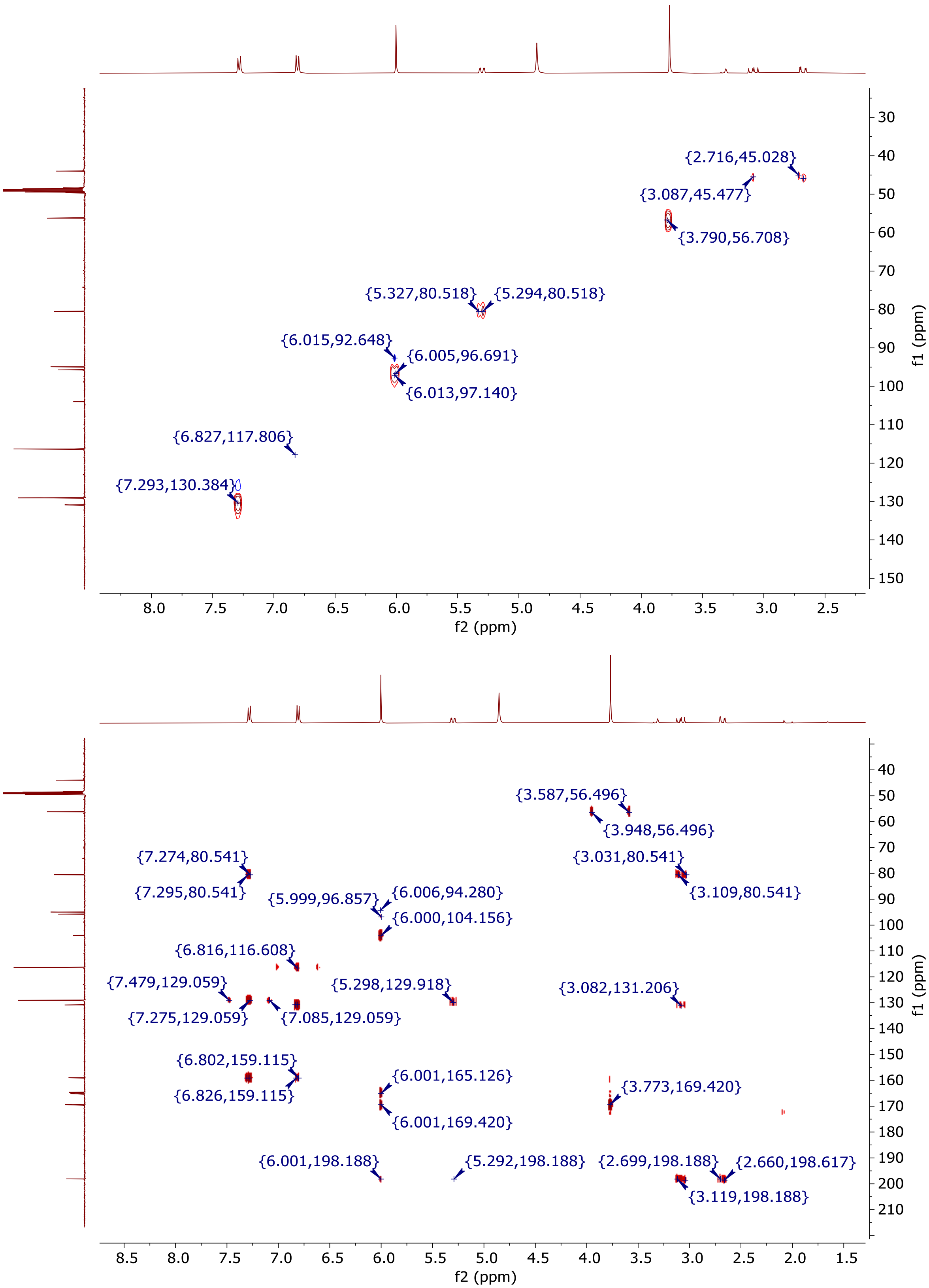

Figure S 2. HSQC and HMBC NMR (MeOD; 400 MHz) of sakuranetin.

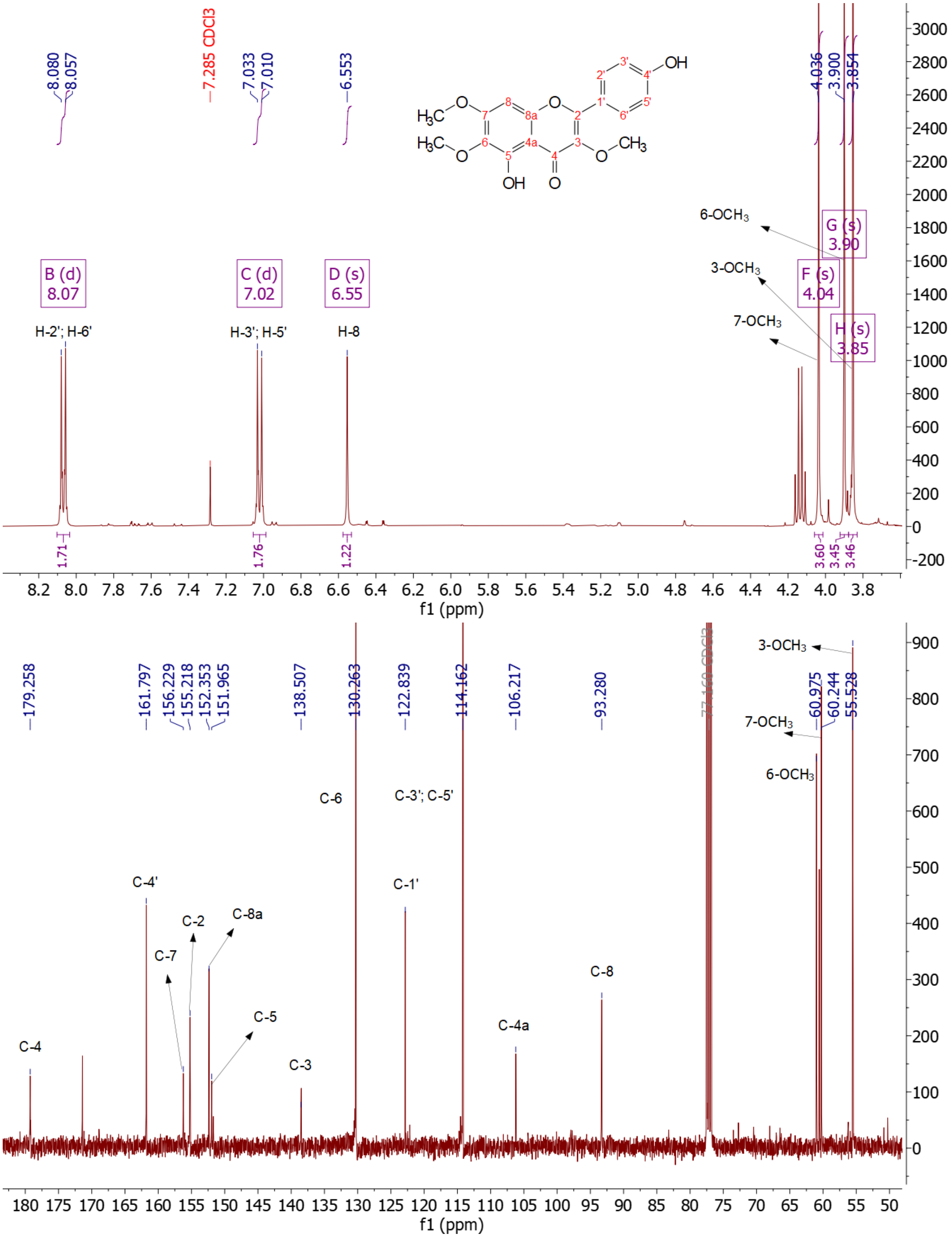

Figure S 3. ^{1}H and ^{13}C NMR ($CDCl_3$; 400 MHz) of penduletin.

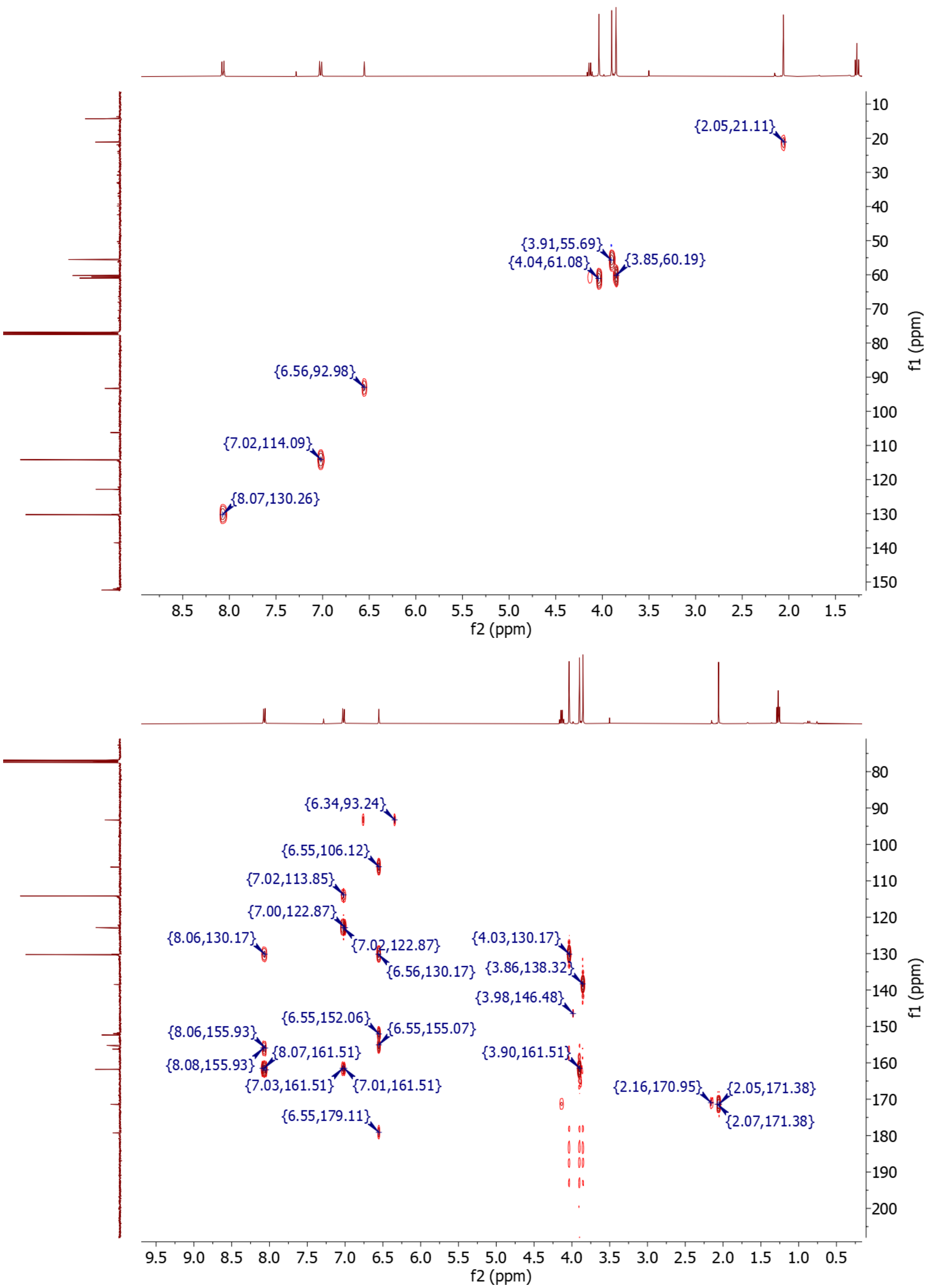

Figure S 4. HSQC and HMBC NMR ($CDCl_3$; 400 MHz) of penduletin.

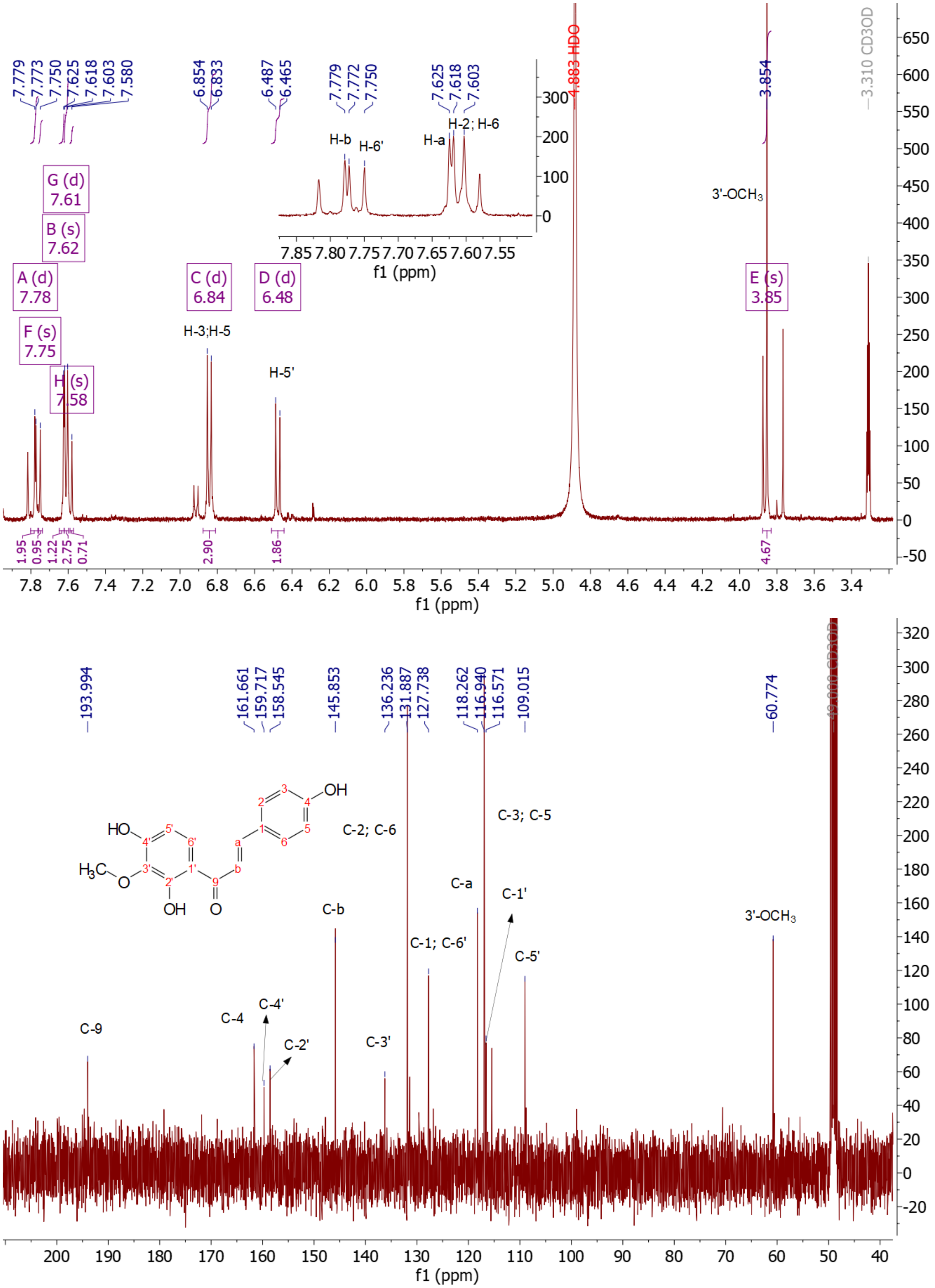

Figure S 5. ^{1}H and ^{13}C NMR (MeOD; 400 MHz) of kukulkanin B.

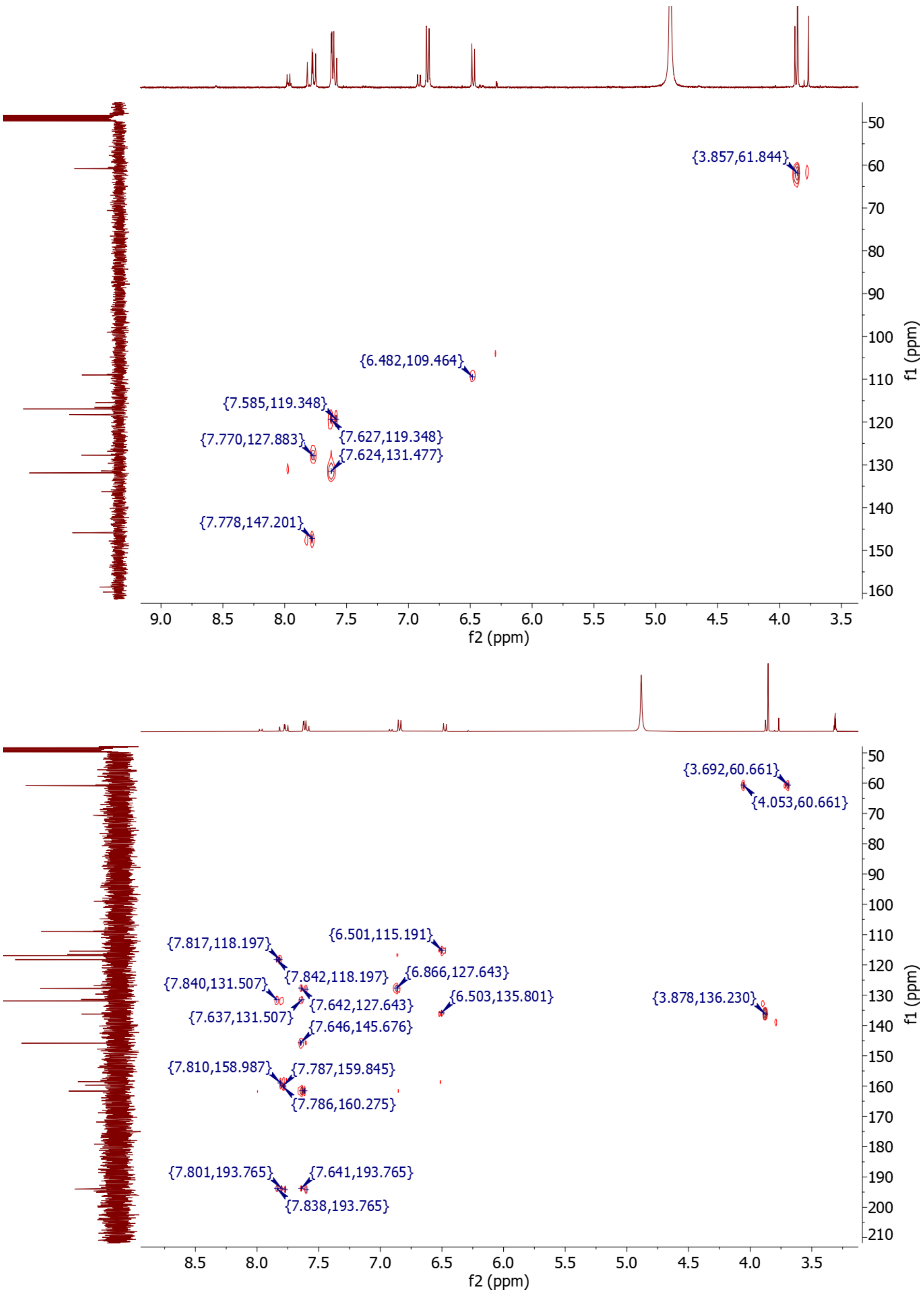

Figure S 6. HSQC and HMBC NMR (MeOD; 400 MHz) of kukulkanin B.

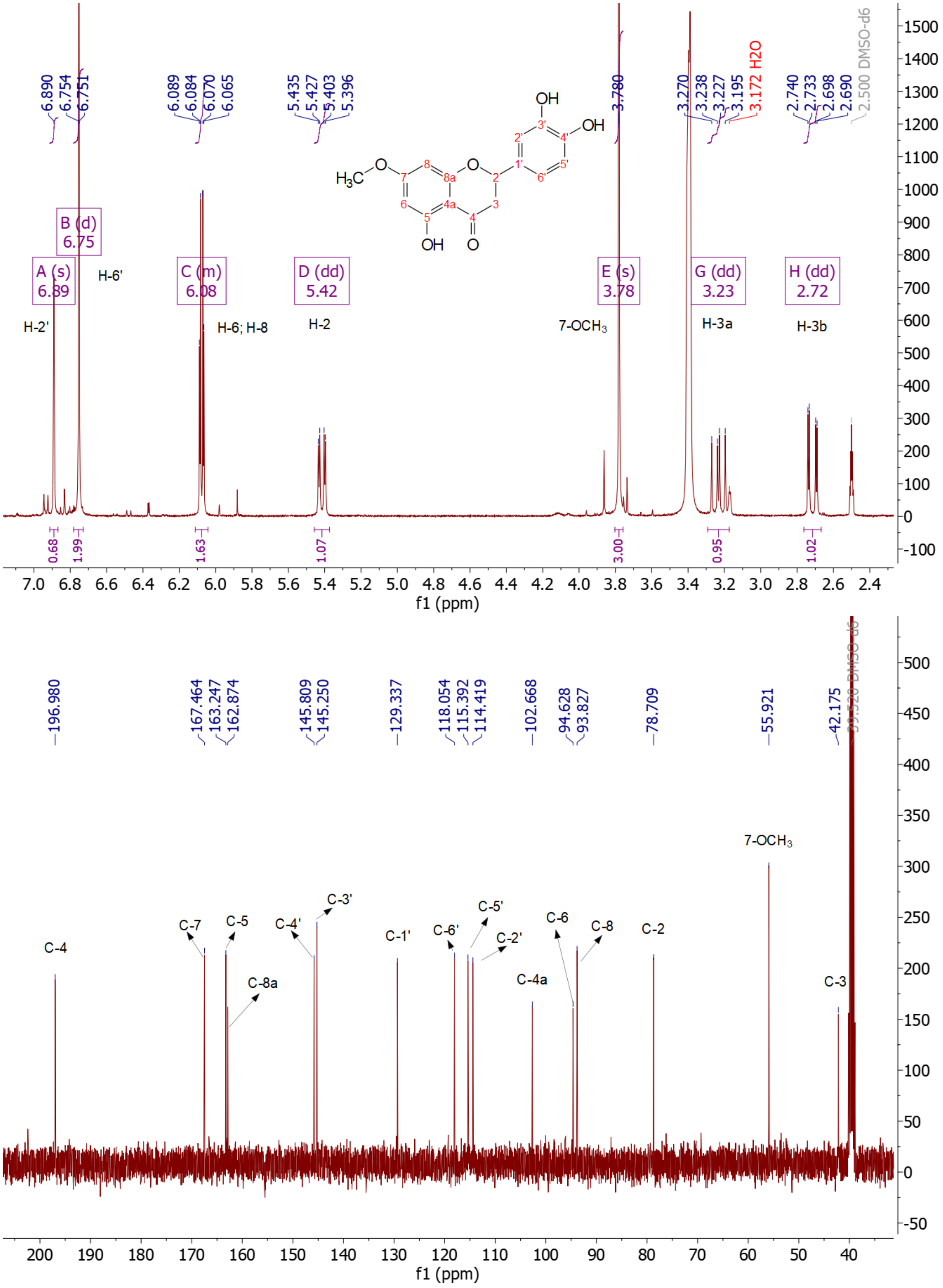

Figure S 7. ^{1}H and ^{13}C NMR (DMSO-*d*; 400 MHz) of 7-*O*-methyleriodictyol.

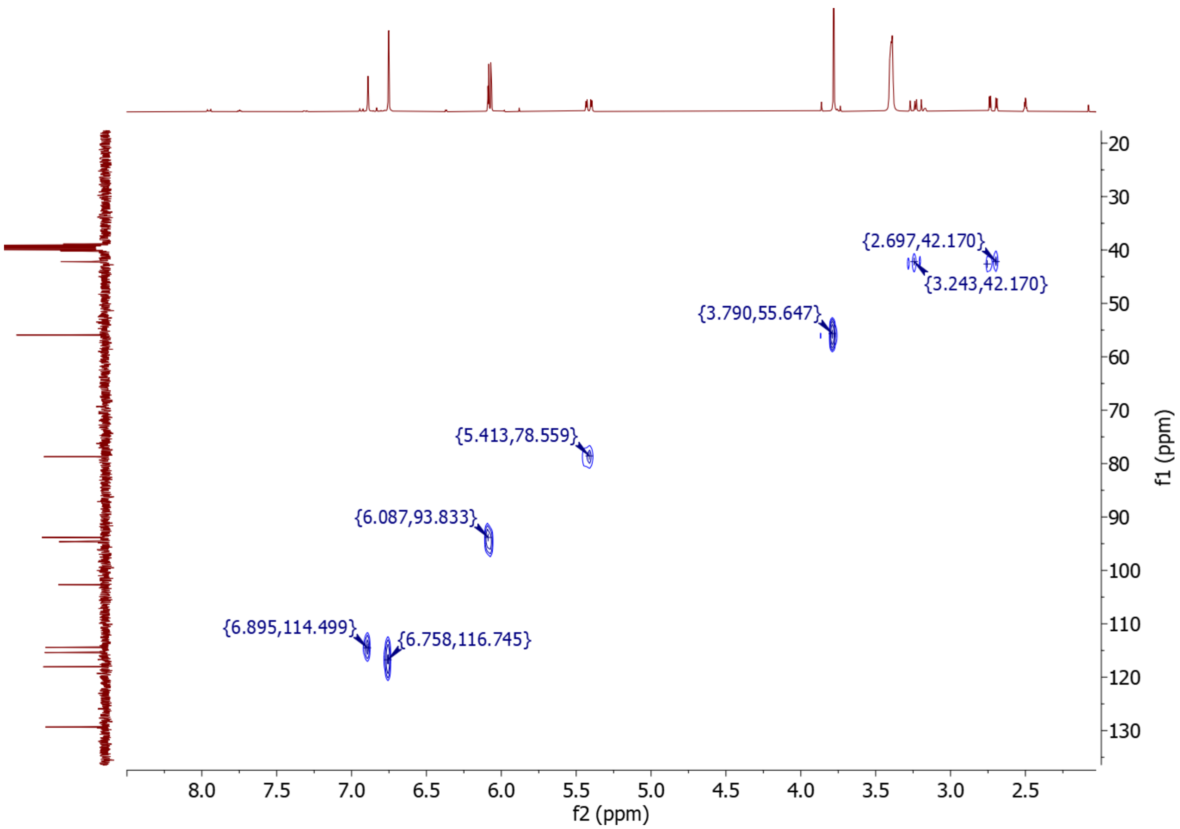

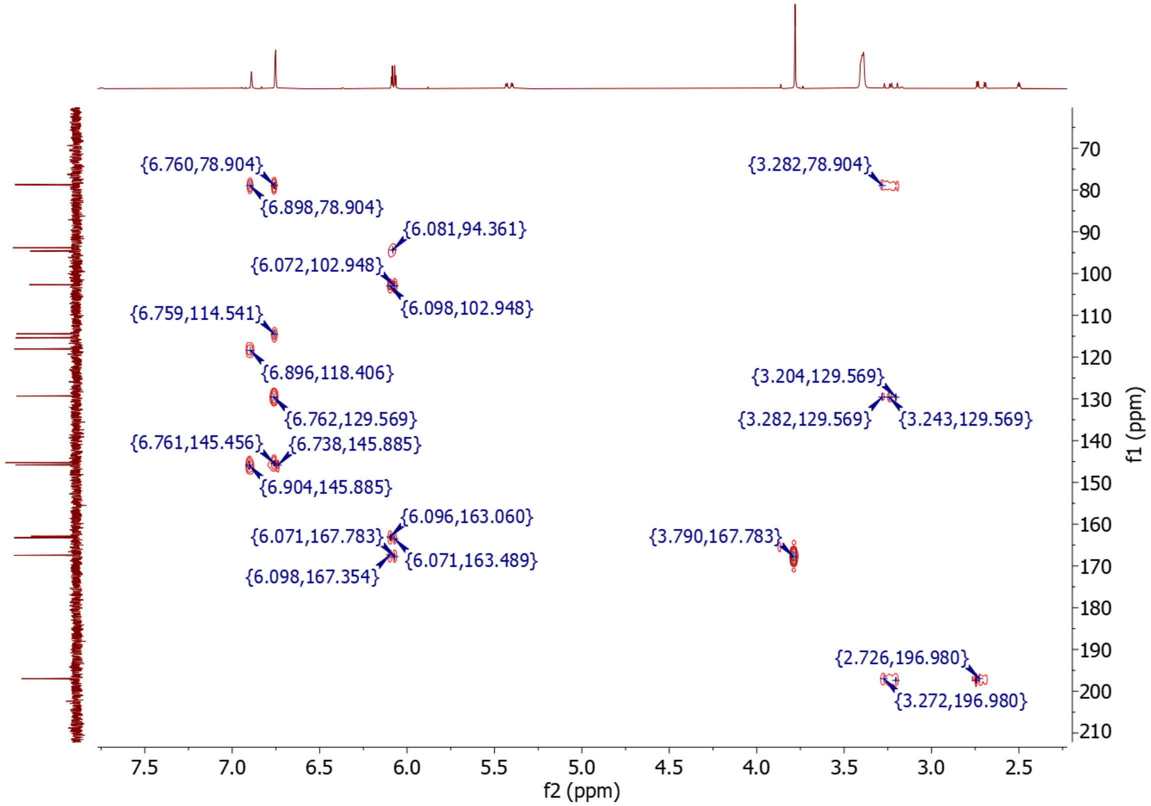

Figure S 8. HSQC and HMBC NMR (DMSO-*d*; 400 MHz) of 7-*O*-methyleriodictyol.

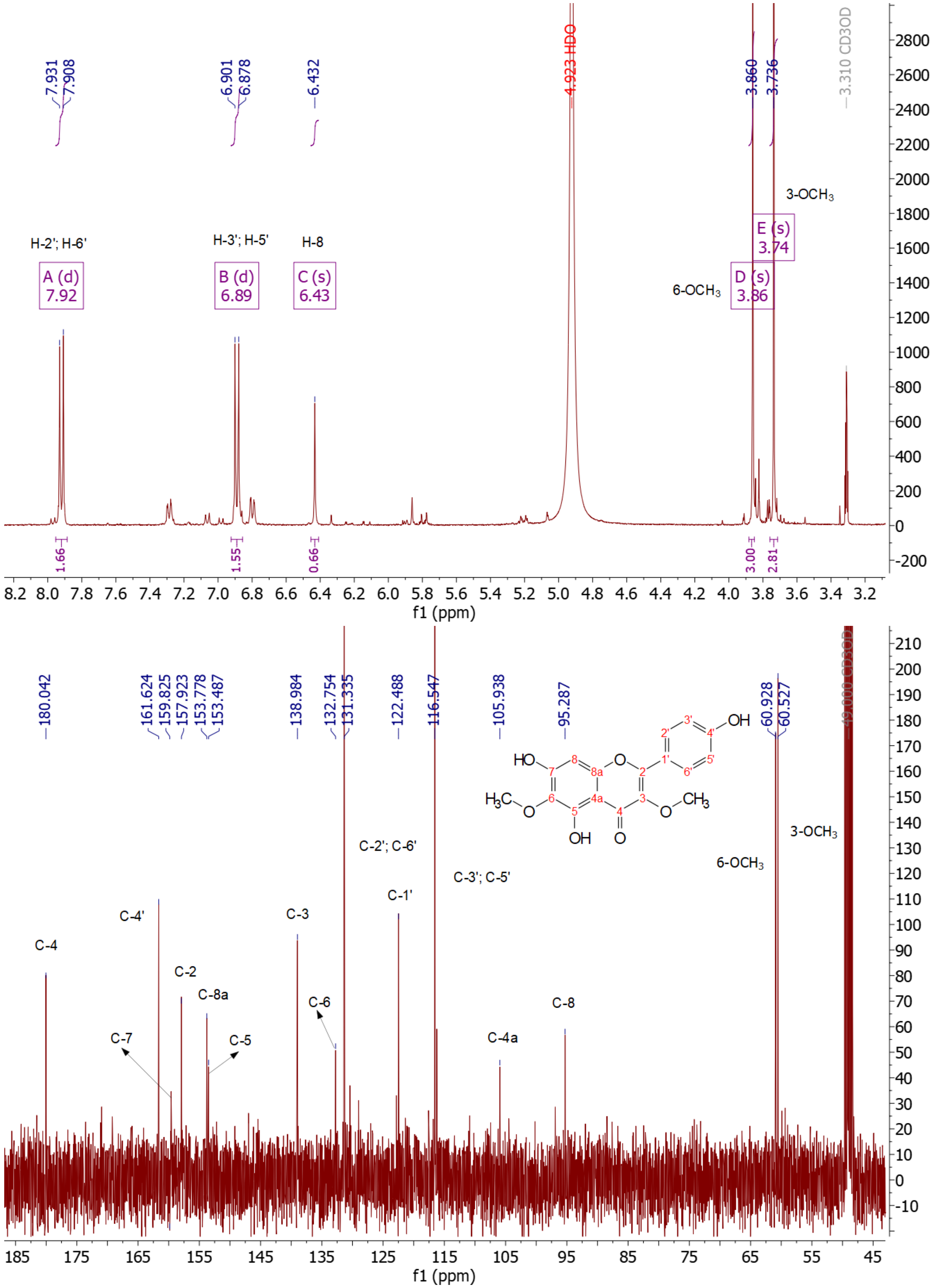

Figure S 9. ^{1}H and ^{13}C NMR (CD_3OD; 400 MHz) of viscosine.

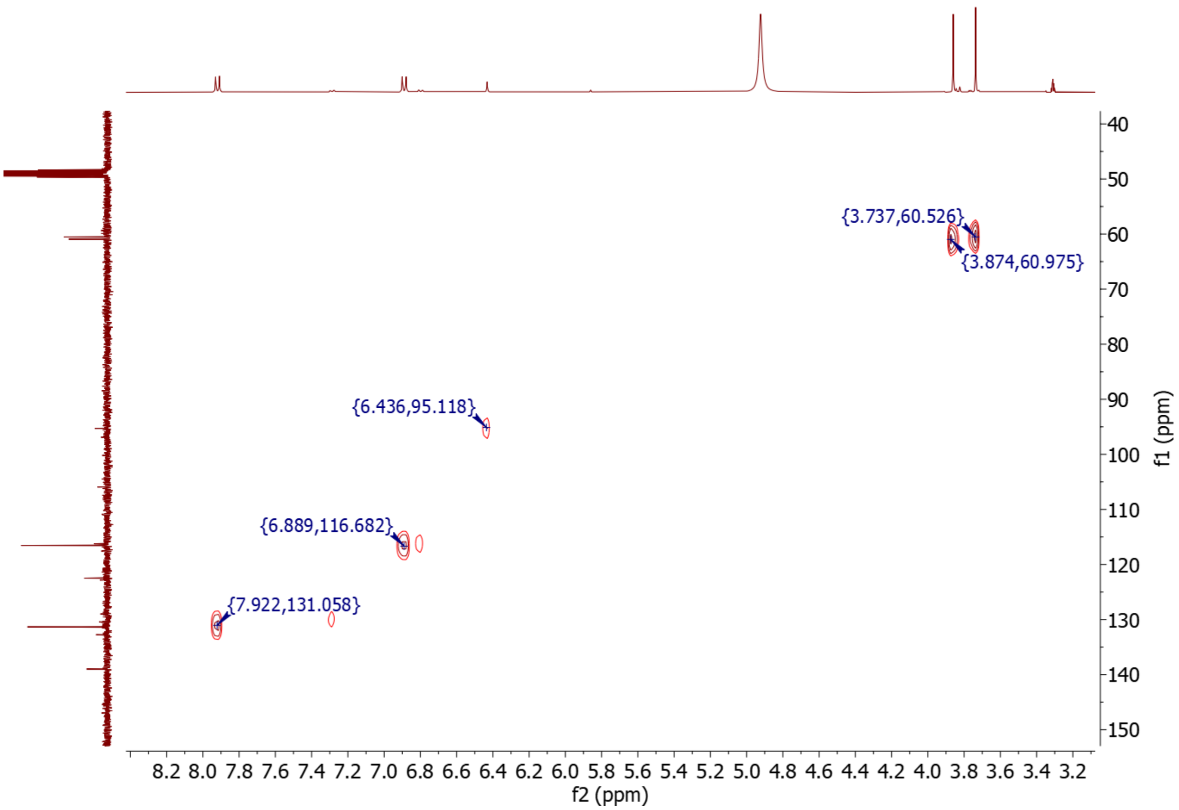

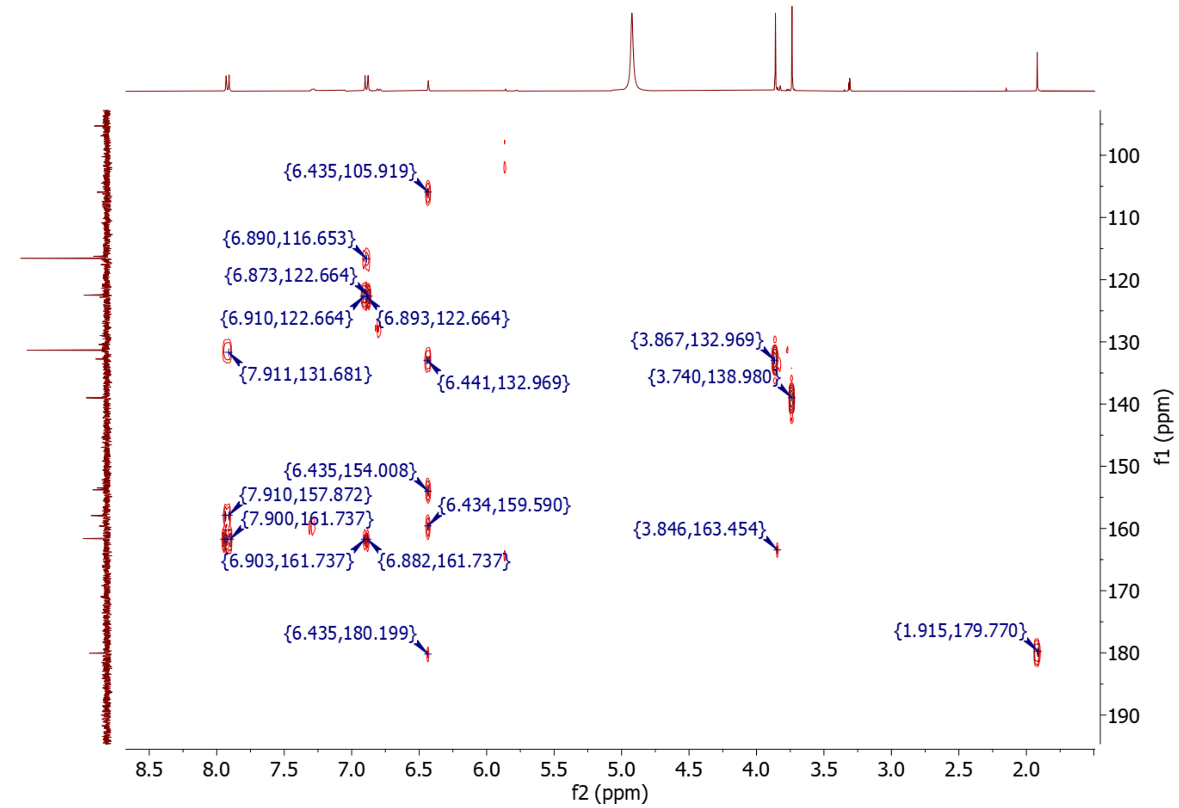

Figure S 10. HSQC and HMBC NMR (CD_3OD; 400 MHz) of viscosine.

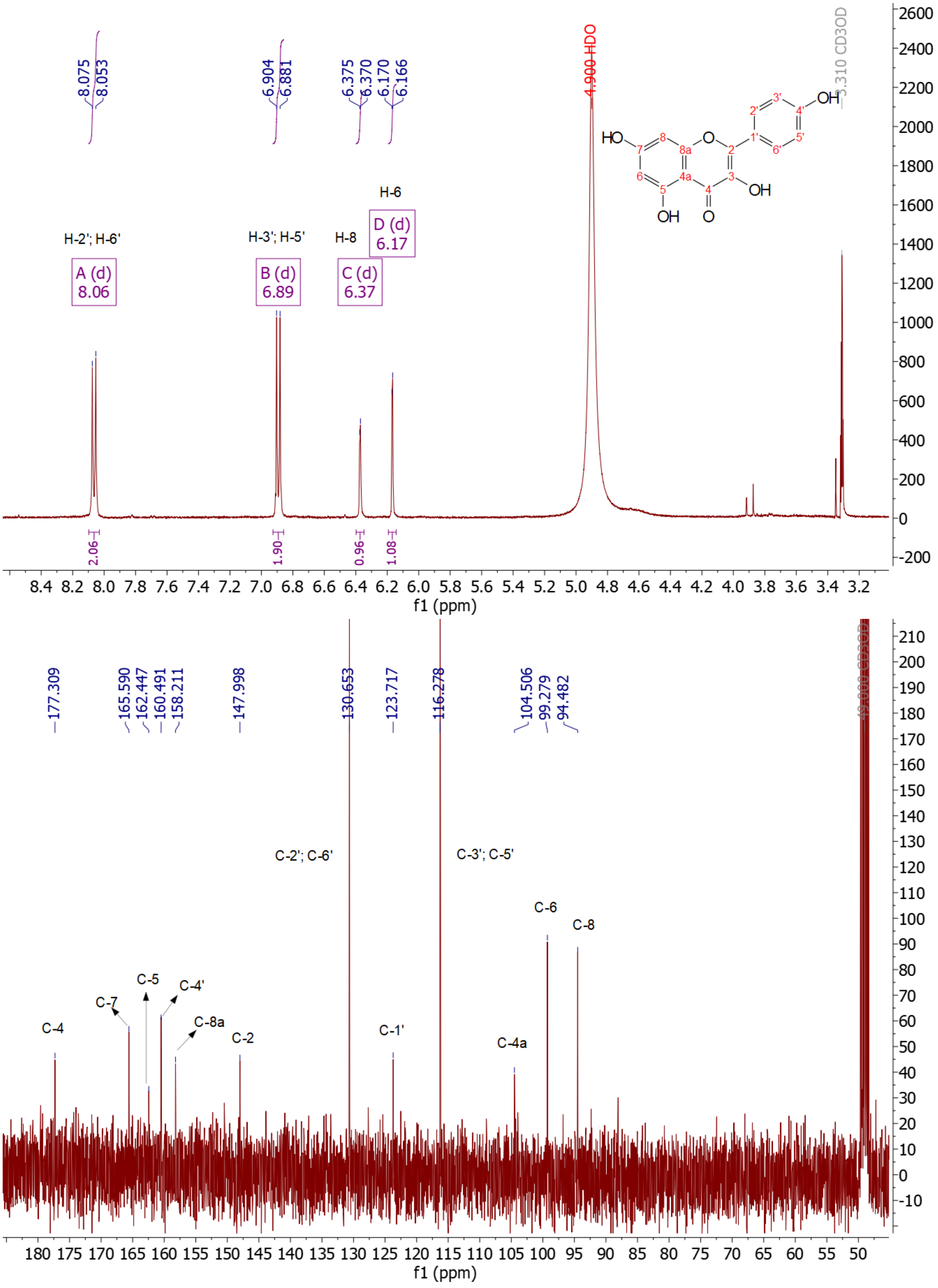

Figure S 11. ^{1}H and ^{13}C NMR (MeOD; 400 M Hz) of kaempferol.

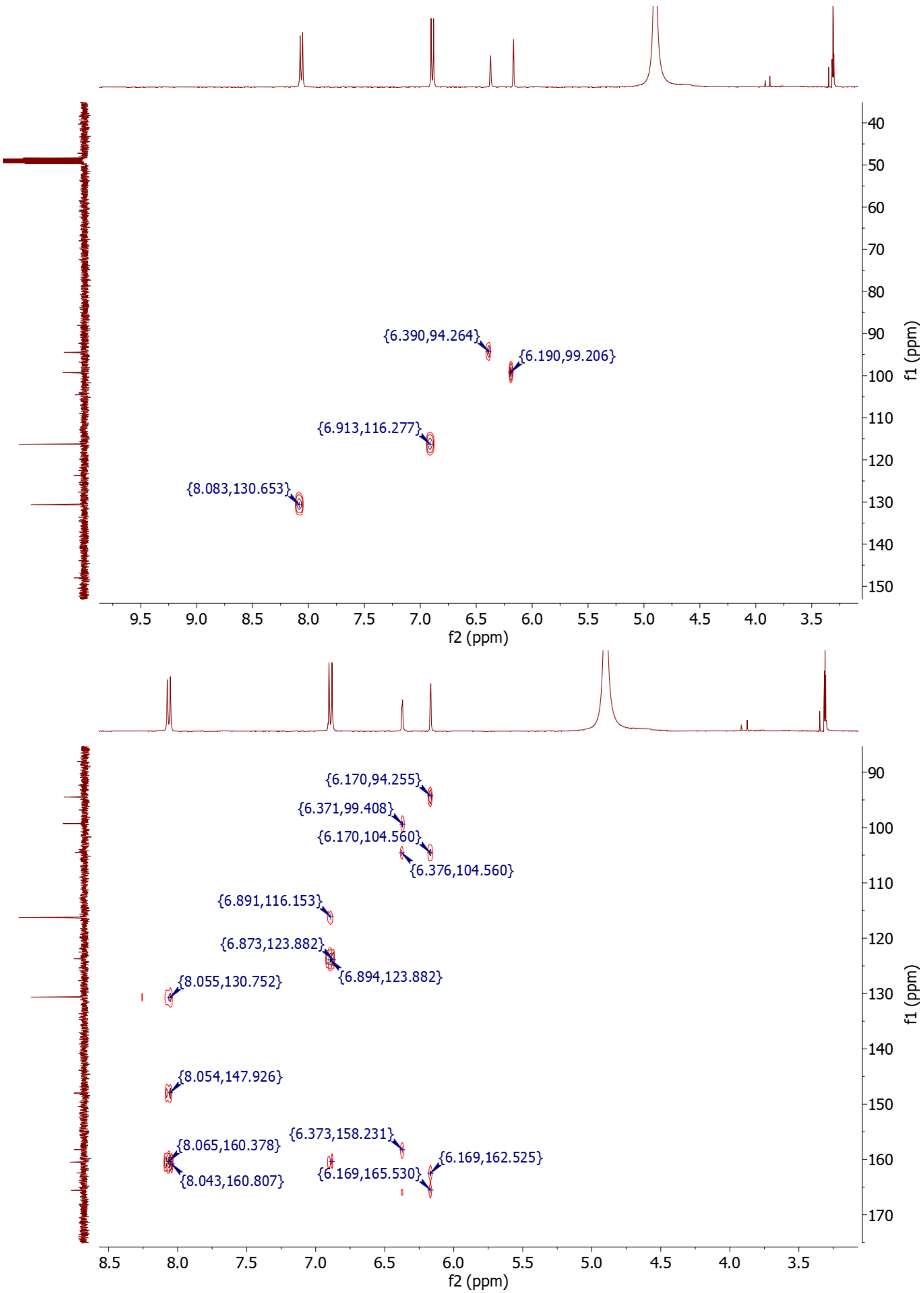

Figure S 12. HSQC and HMBC NMR (MeOD; 400 MHz) of kaempferol.

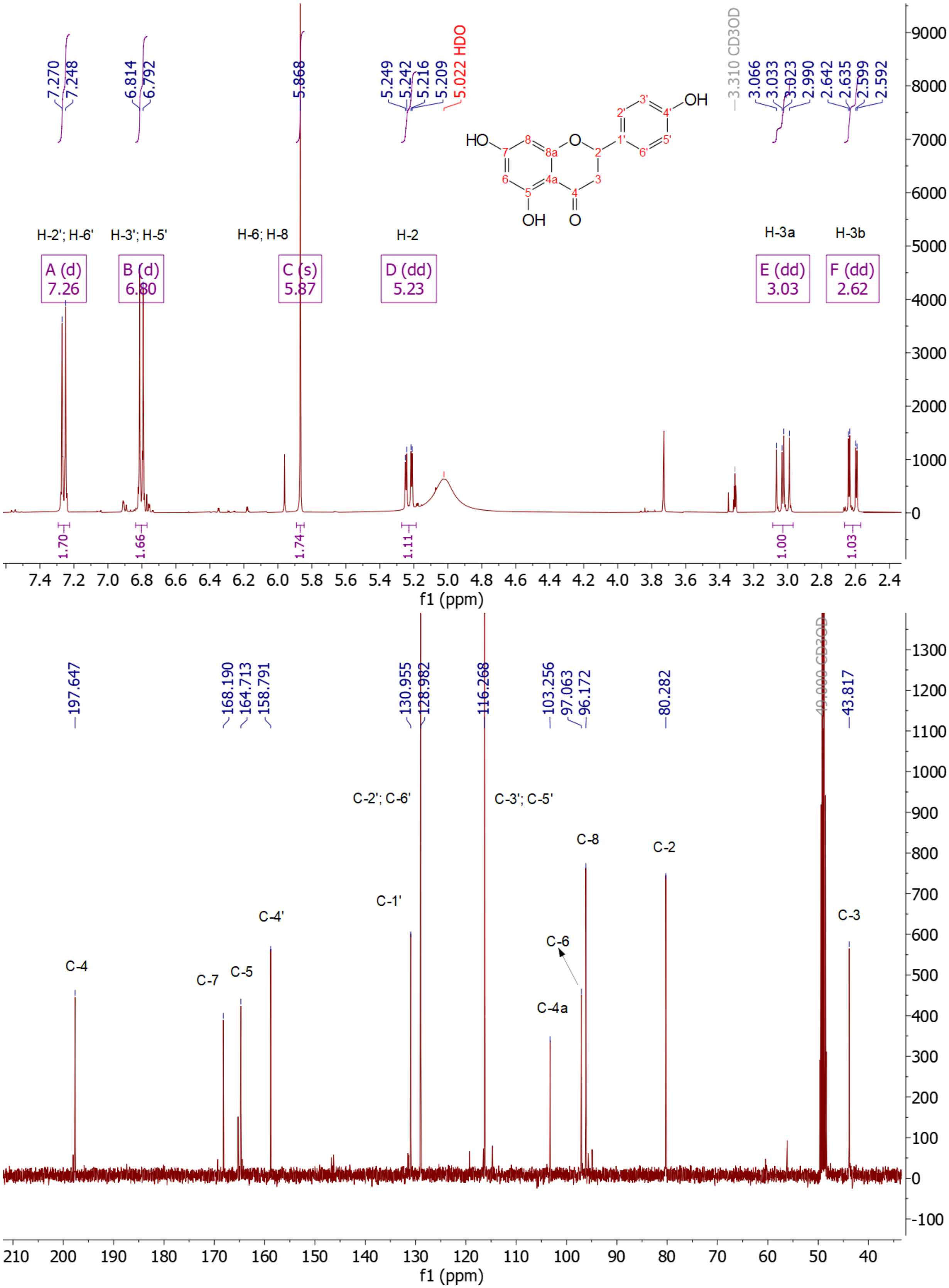

Figure S 13. ^{1}H and ^{13}C NMR (MeOD; 400 MHz) of naringenin.

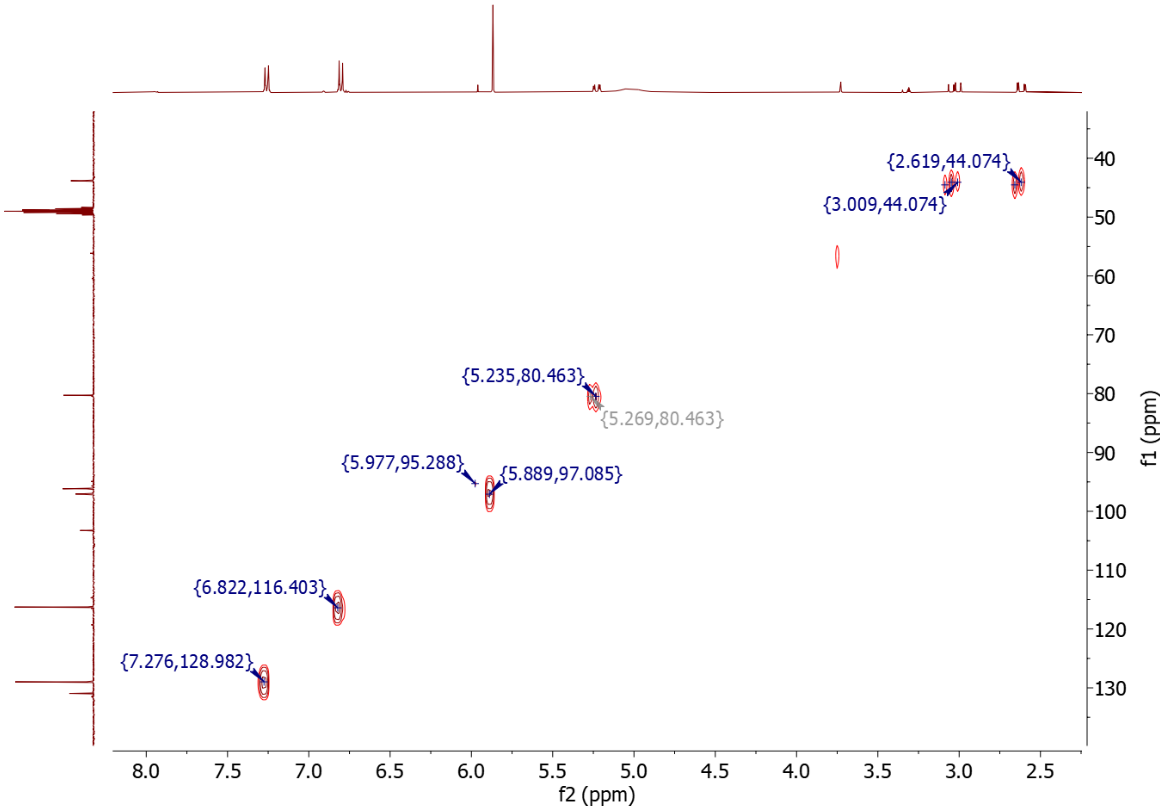

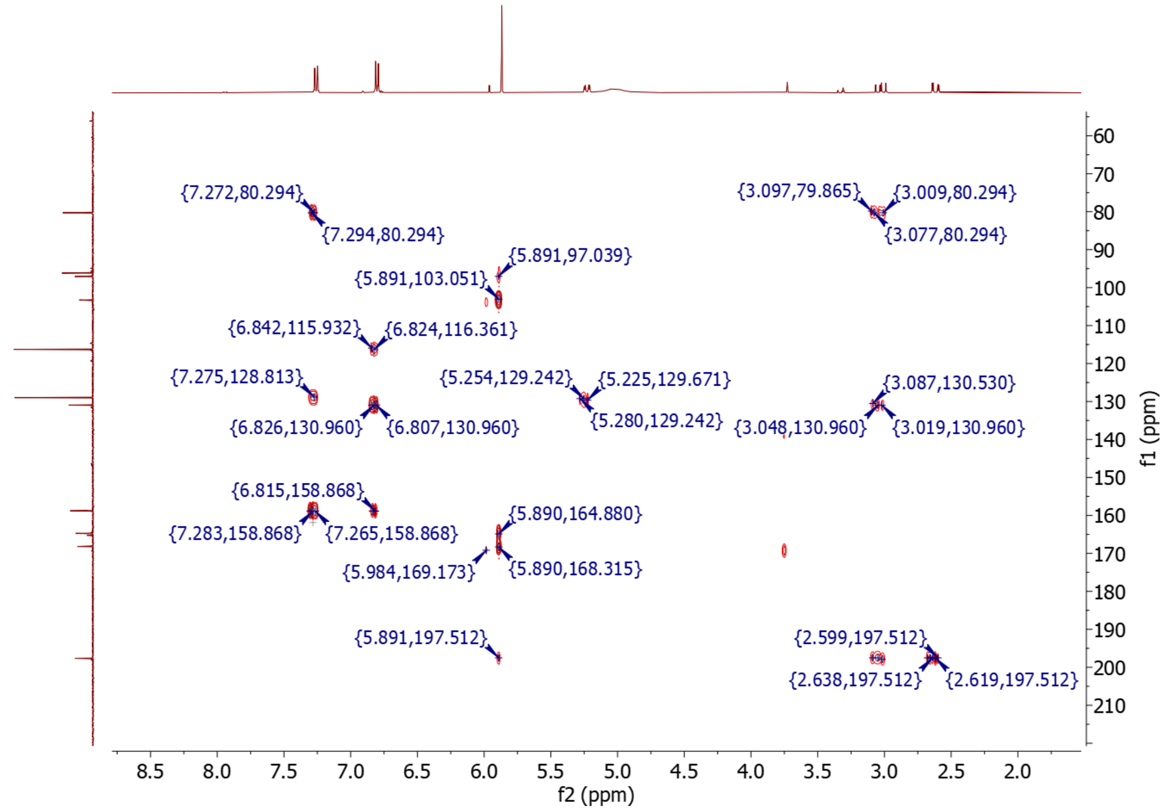

Figure S 14. HSQC and HMBC NMR (MeOD; 400 MHz) of naringenin.

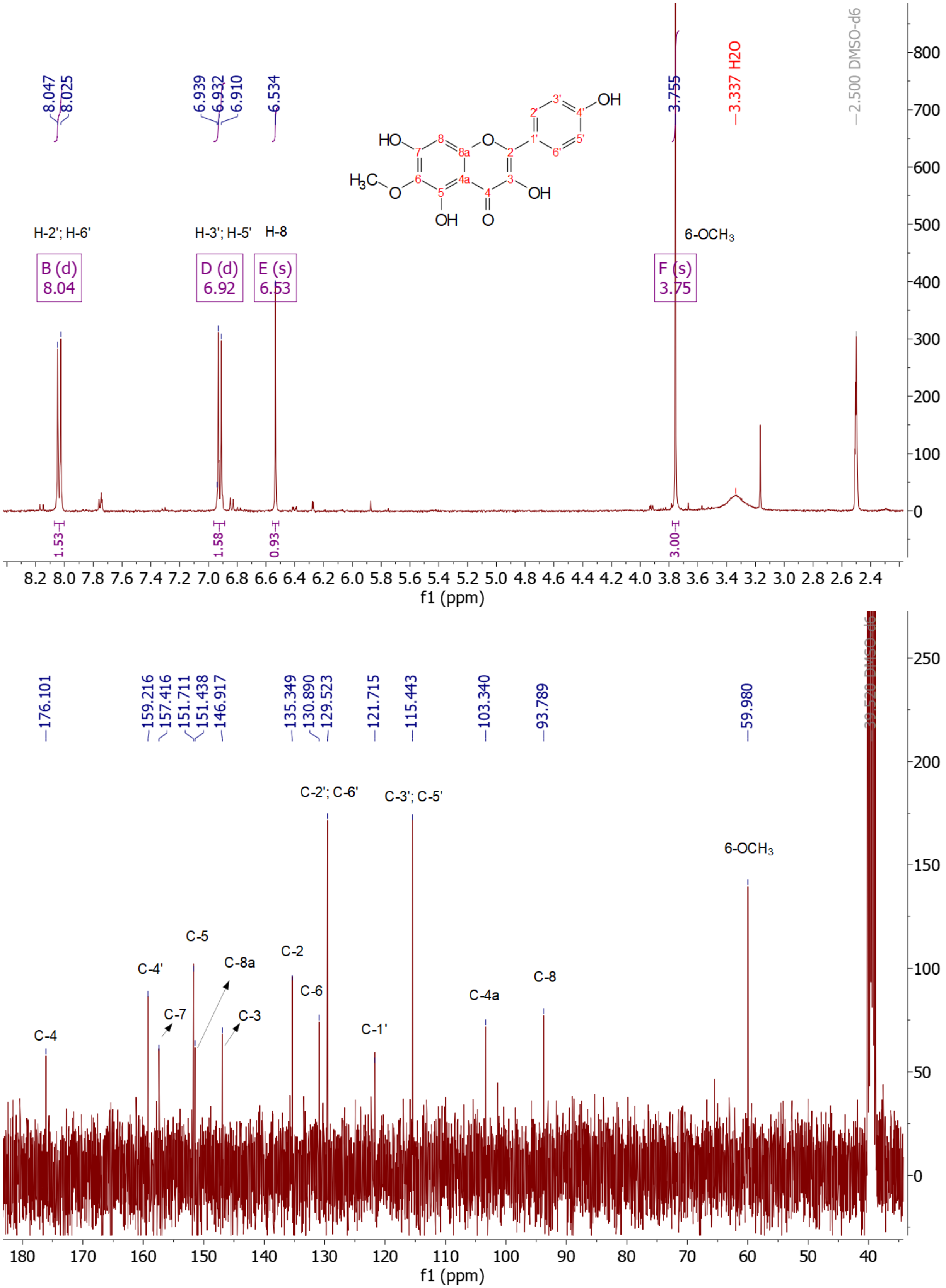

Figure S 15. ^{1}H and ^{13}C NMR (DMSO-*d*; 400 MHz) of 6-methoxykaempferol.

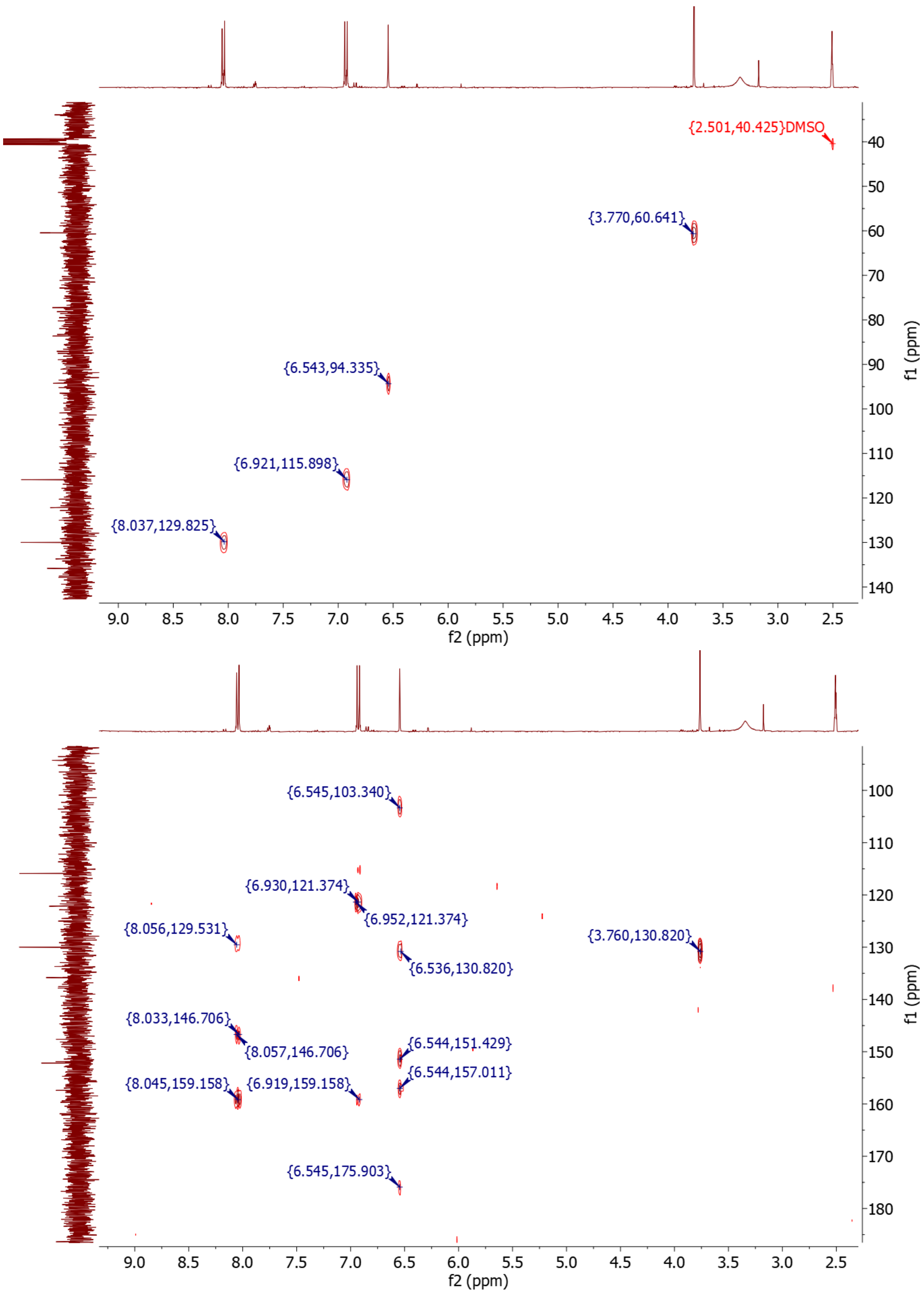

Figure S 16. HSQC and HMBC NMR (DMSO-*d*; 400 MHz) of 6-methoxykaempferol.

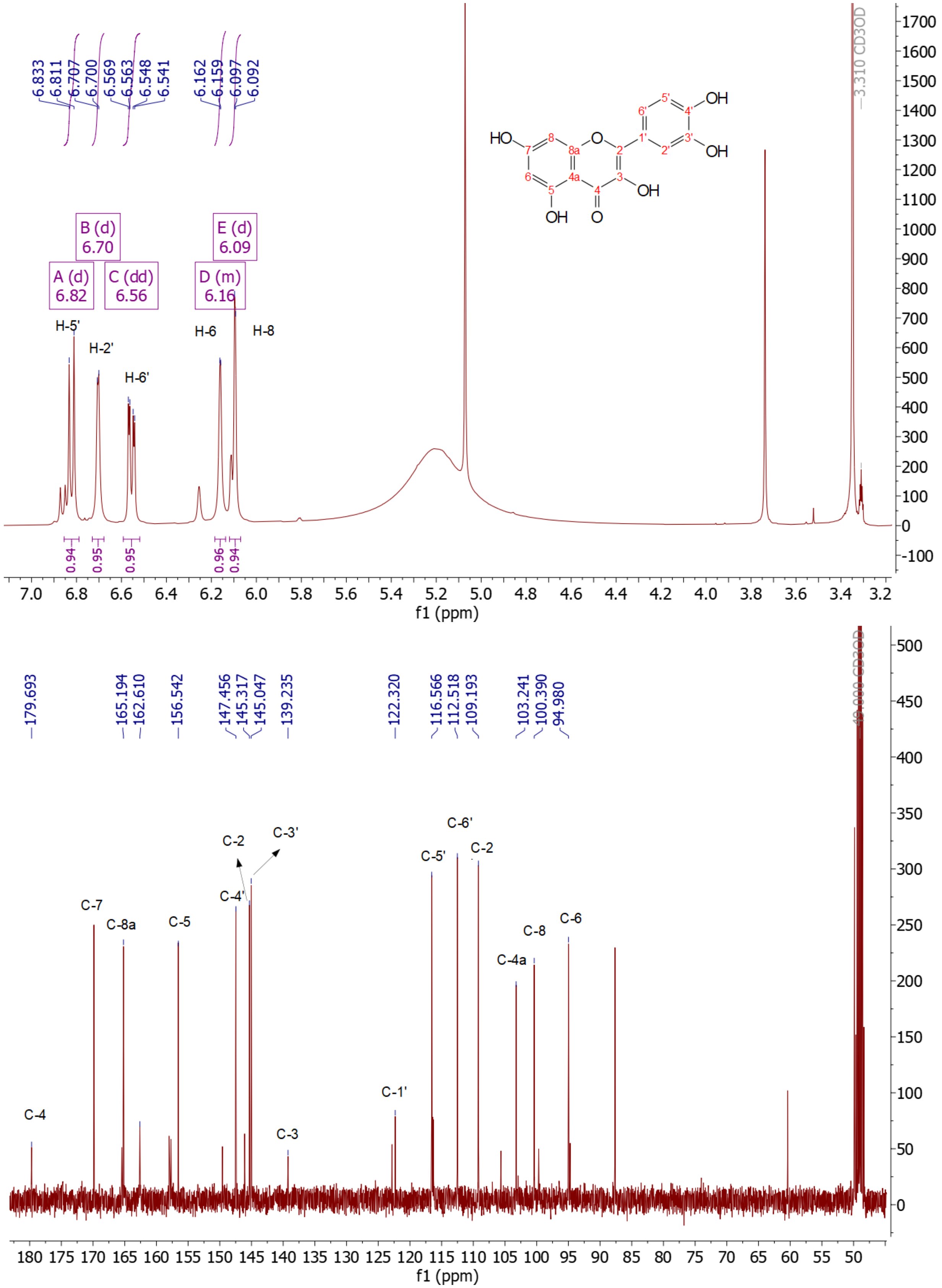

Figure S 17. ^{1}H and ^{13}C NMR (MeOD; 400 MHz) of quercetin.

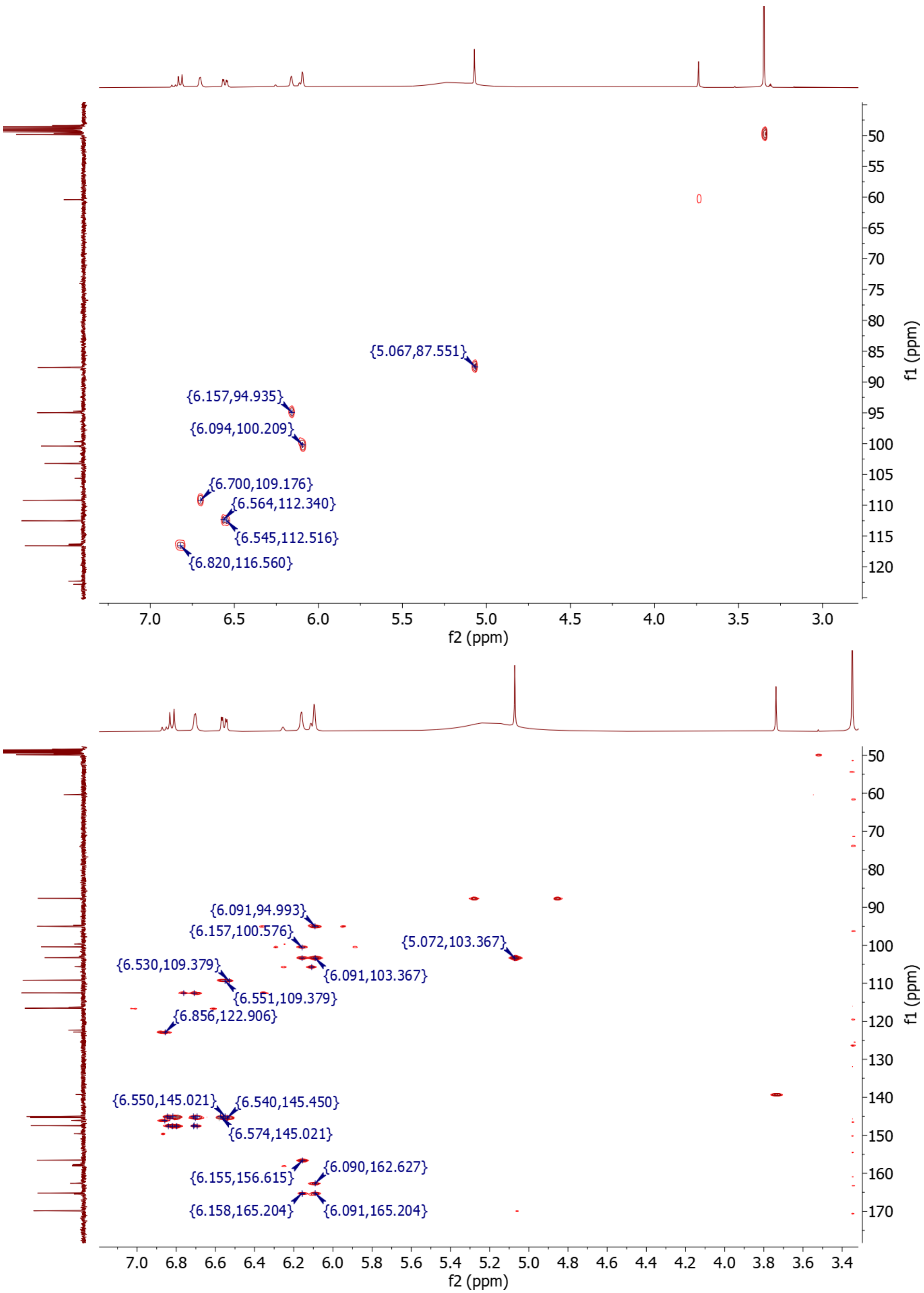

Figure S 18. HSQC and HMBC NMR (MeOD; 400 MHz) of quercetin.

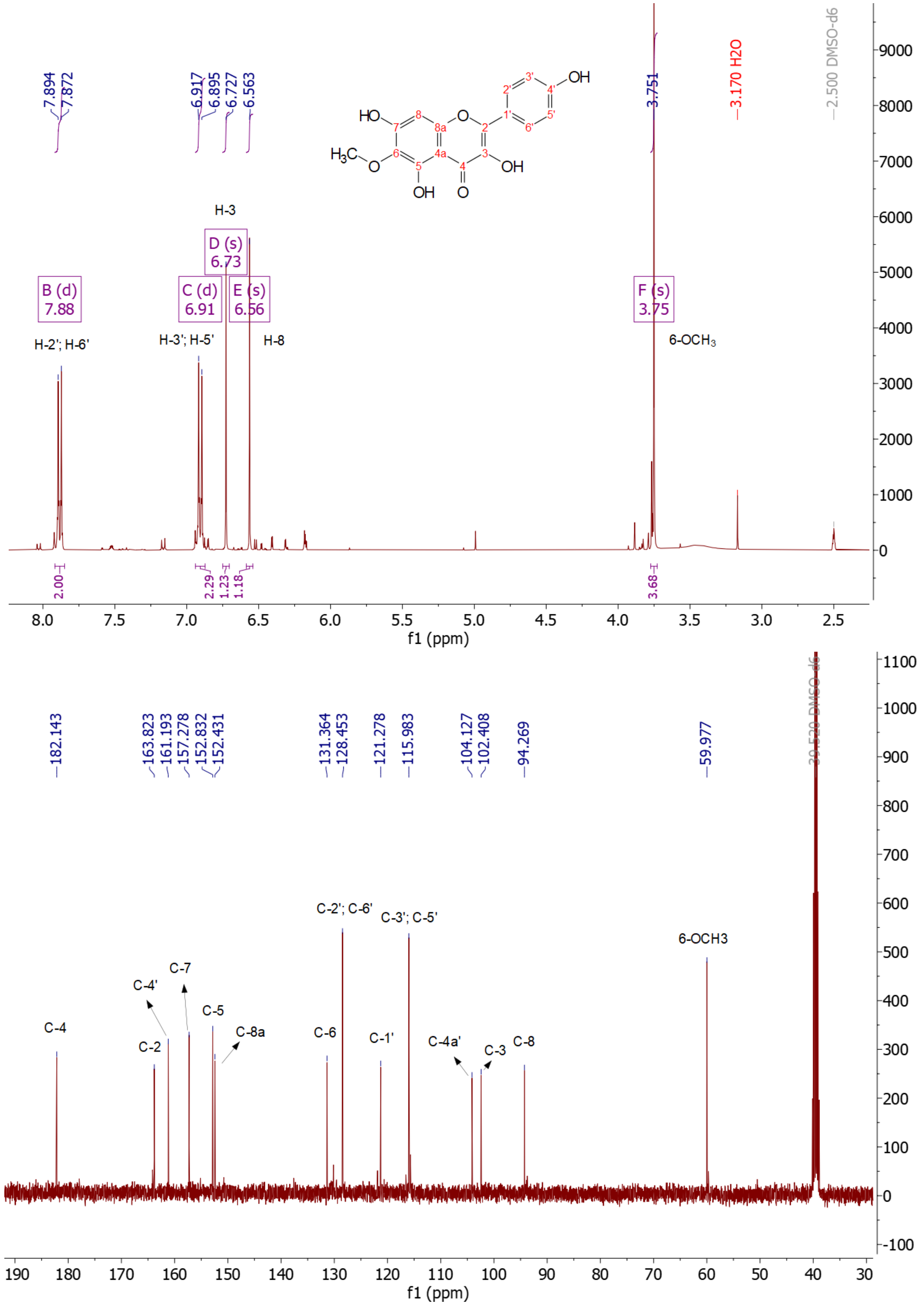

Figure S 19. ^{1}H and ^{13}C NMR (DMSO-*d*; 400 MHz) of hispidulin.

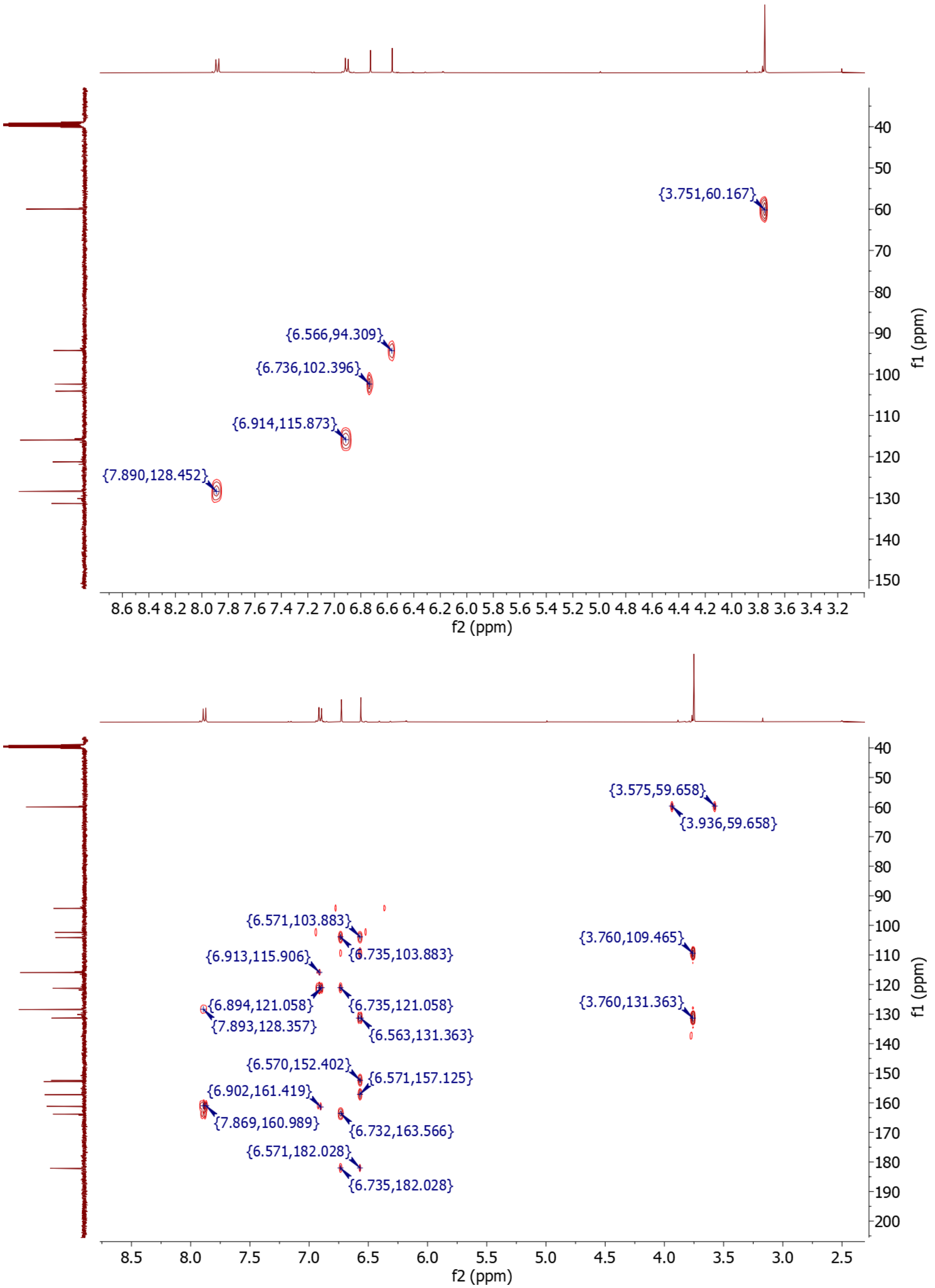

Figure S 20. HSQC and HMBC NMR (DMSO-*d*; 400 MHz) of hispidulin.

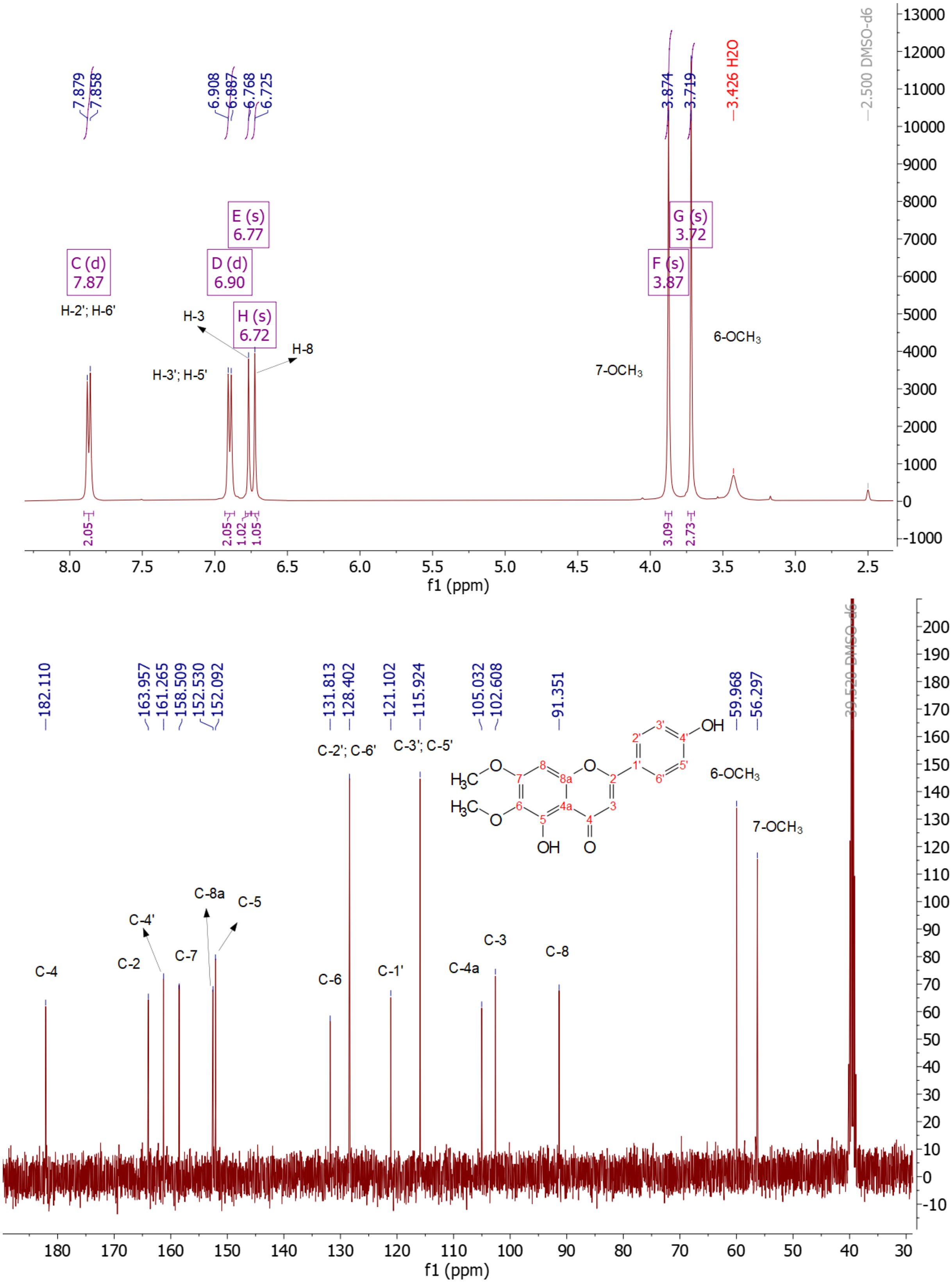

Figure S 21. ^{1}H and ^{13}C NMR (MeOD; 400 MHz) of cirsimaritin.

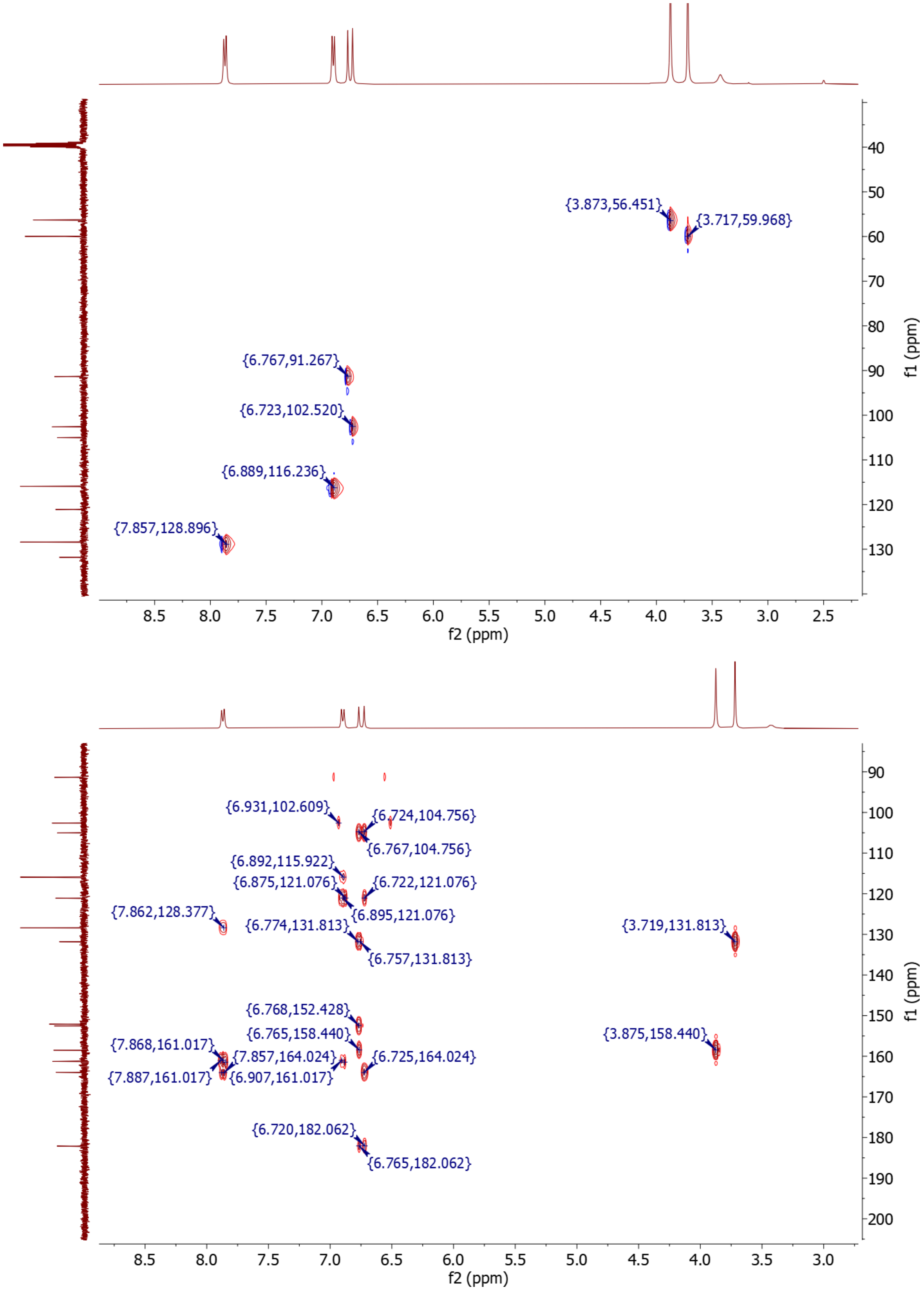

Figure S 22. HSQC and HMBC NMR (MeOD; 400 MHz) of cirsimaritin.

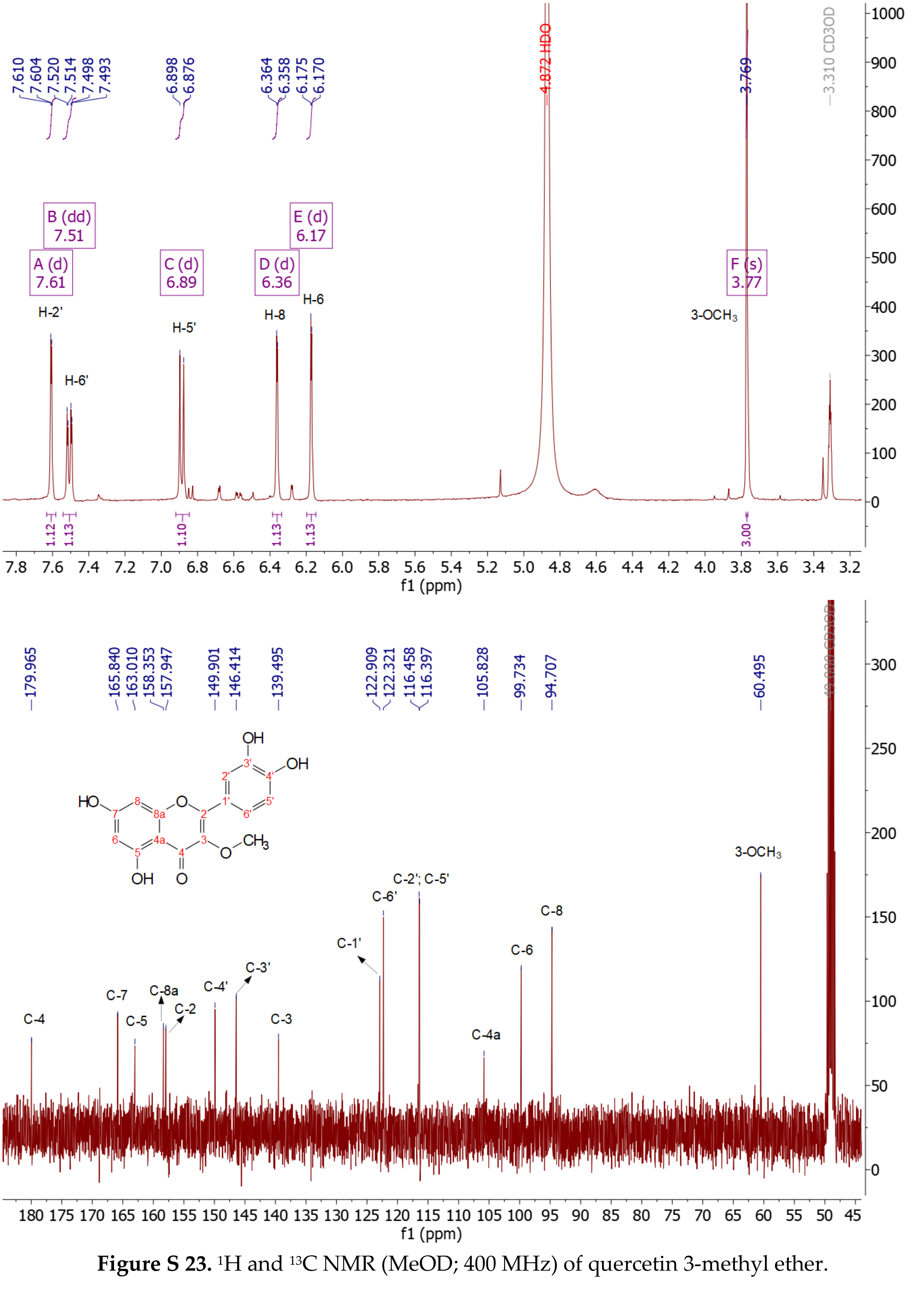

Figure S 23. ^{1}H and ^{13}C NMR (MeOD; 400 MHz) of quercetin 3-methyl ether.

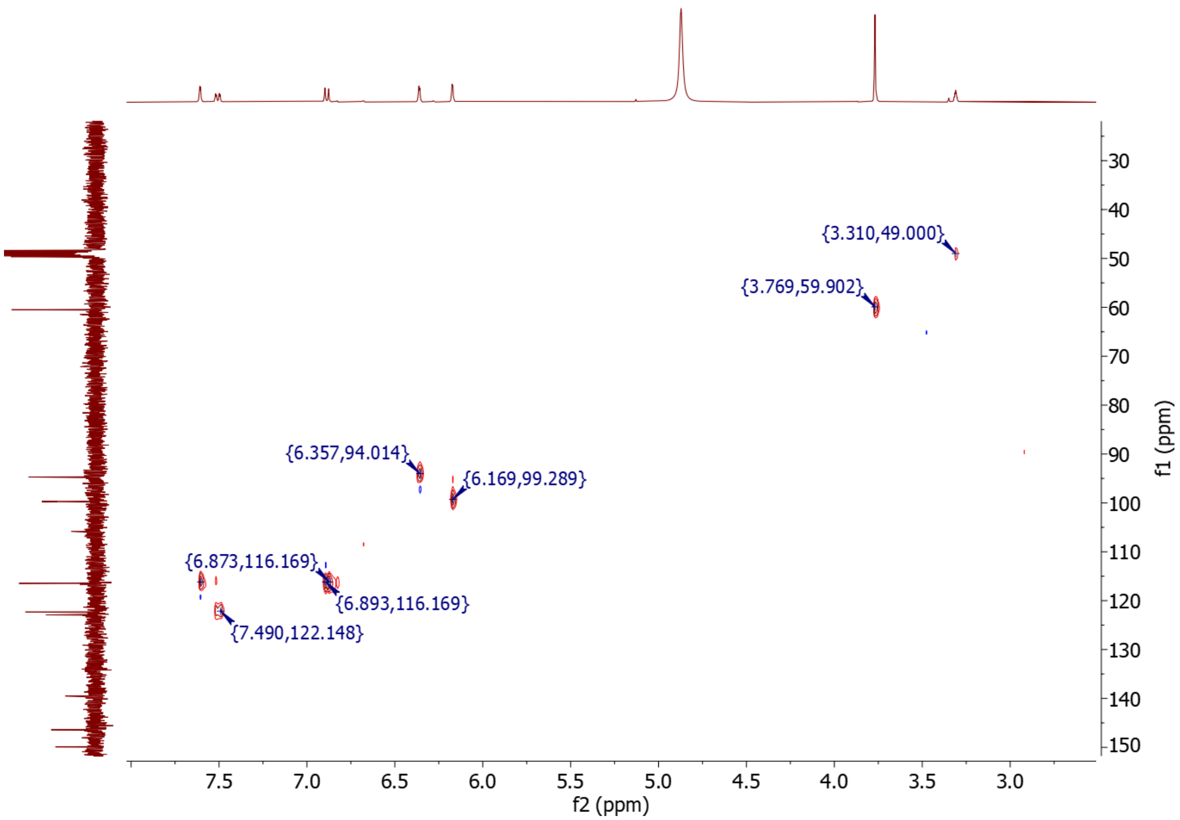

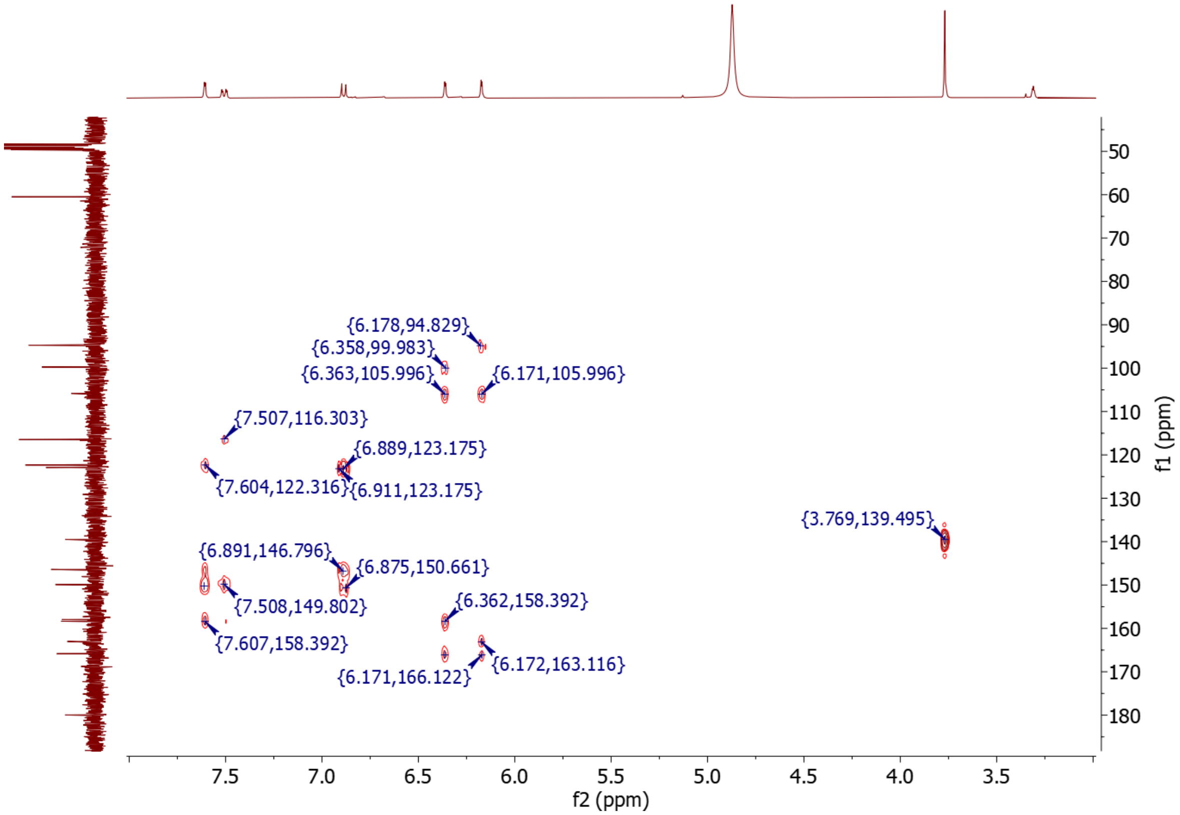

Figure S 24. HSQC and HMBC NMR (MeOD; 400 MHz) of quercetin 3-methyl ether.
